# The Transcription Factors NFYA1 and GBF3 Jointly Regulate *CHS2* to Promote Tangeretin Accumulation and Cold Tolerance in *Citrus*

**DOI:** 10.1111/pbi.70371

**Published:** 2025-09-16

**Authors:** Peng Xiao, Jing Qu, Yike Zeng, Wei Xiao, Tian Fang, Yilei Wang, Xi Zeng, Chunlong Li, Ji‐Hong Liu

**Affiliations:** ^1^ National Key Laboratory for Germplasm Innovation & Utilization of Horticultural Crops, College of Horticulture and Forestry Sciences Huazhong Agricultural University Wuhan China; ^2^ Hubei Hongshan Laboratory Wuhan China

**Keywords:** *Citrus ichangensis*, cold stress, GBF3, NFYA1, tangeretin, transcriptional cascade

## Abstract

Tangeretin has been known as a polymethoxylated flavone conferring both phytoprotection and nutraceutical value, but cryoprotective roles and molecular regulation of tangeretin under abiotic stresses remain largely unexplored. In this study, we demonstrated that cold treatment led to greater accumulation of tangeretin and upregulation of *Chalcone Synthase 2 (CiCHS2)* in Ichang papeda (*Citrus ichangensis*), a cold‐hardy citrus species, relative to a cold‐sensitive genotype. CiCHS2, localised in the endoplasmic reticulum, was shown to function in cold tolerance by modulating tangeretin synthesis. We revealed that the transcription factors CiNFYA1 and CiGBF3 act as transcriptional activators of *CiCHS2* by interacting with the CCAAT and G‐box (CACGTG) elements, respectively, in the gene promoter. Furthermore, CiNFYA1 could regulate *CiGBF3* by binding to the gene promoter. In addition, CiNFYA1 physically interacted with CiGBF3, and the resulting protein complex further promoted transactivation of *CiCHS2*. Moreover, CiNFYA1 and CiGBF3 were demonstrated to play a positive role in the modulation of cold tolerance by regulating *CHS2*‐mediated tangeretin accumulation. Taken together, our findings unravel a hierarchical regulatory network wherein CiNFYA1‐CiGBF3 cooperatively activated *CiCHS2*‐dependent tangeretin biosynthesis in response to cold stress. These results advance our understanding of the molecular regulation of tangeretin accumulation under cold stress and provide valuable targets for engineering cold‐tolerant crops through metabolic engineering.

## 1 Introduction

Given the intensification of global climate change, plants are frequently threatened by various environmental constraints, among which cold stress has been demonstrated to exert serious damage to plant growth, development, geographic distribution and crop yield (Zhu 2016; Zhang, Zhu, Gong, and Zhu 2022; Ding et al. 2024; Zeng et al. 2025). Therefore, elevation of cold tolerance is crucial for safeguarding food security and advancing sustainable agricultural practices. In nature, plants have evolved integrated and sophisticated defence strategies ranging from signal perception, transcriptional reprogramming, to physiological and biochemical alteration to perceive, adapt and counteract the environmental stresses (Fryer et al. 1998; Zhao et al. 2015; Guo et al. 2018; Dahro et al. 2023). Accumulating evidence indicates that stress‐responsive genes play crucial roles in the plant's acclimation to abiotic stresses (Ding and Yang 2022; Kidokoro et al. 2022; Ding et al. 2024; Zeng et al. 2025). It is thus conceivable that elucidation of the molecular basis of stress adaptation and acclimation is pressing and essential for identifying core genes and their products that are closely associated with stress tolerance.

A substantial body of work has demonstrated that plants, when exposed to abiotic stresses, synthesise a variety of protective substances that function in coping with the stress conditions by efficiently detoxifying reactive oxygen species (ROS), maintaining cell membrane fluidity and adjusting osmotic pressure (Khan et al. 2021; Hoermiller et al. 2022; Wang, Zuo, et al. 2022; John et al. 2024; Huang, Zhang, et al. 2025; Meng et al. 2025; Qu et al. 2025). Some of these compounds are referred to as osmolytes that work synergistically to sustain optimum osmotic homeostasis, leading to stabilisation of cellular membrane structures and enhanced cold tolerance. It has been widely proposed that except for the activation of key antioxidative enzymes, including superoxide dismutase (SOD) and peroxidase (POD), substantial accumulation of nonenzymatic antioxidants is crucial for maintaining redox homeostasis in plants under stressful conditions (Xiao, Qu, et al. 2024; Zhang et al. 2024; Guo et al. 2025). Flavonoids represent a prominent group of these protective compounds that exhibit an essential role in ROS scavenging through electron donation and metal chelation mechanisms (Calderon Flores et al. 2021; Bao et al. 2025; Li et al. 2025). Tangeretin (4′,5,6,7,8‐pentamethoxyflavone), one of the flavonoids, is predominantly present in fruit peels and leaves of citrus that has been known for its pharmaceutical properties and nutraceutical applications (Guo et al. 2021; Yang et al. 2021; Liu et al. 2024). By contrast, the functional roles of tangeretin in plant stress tolerance remain poorly understood.

Flavonoid synthesis is initiated by chalcone synthase (CHS) that catalyses the conversion of *p*‐coumaroyl‐coenzyme A into chalcone, which is then integrated into metabolic fluxes to form other flavonoids, including tangeretin (Shirley et al. 1995; Tohge et al. 2013). Increasing evidence has identified CHS as a rate‐limiting gatekeeper enzyme responsible for flavonoid production under abiotic stresses (Dong and Lin 2021; Luo et al. 2024). Overexpression of *CHS* has been shown to increase the levels of endogenous flavanols and improve the salt tolerance of rice, poplar and rape (Cui et al. 2014; Kim et al. 2017; Song et al. 2024). In addition, *CHS* has been revealed to participate in the modulation of anthocyanin biosynthesis in apple and strawberry under cold stress (Mei et al. 2023; Luo et al. 2024). Besides, several transcription factors (TFs) that regulate *CHS* expression in response to drought stress have been unveiled. For example, WRKY17 and WRKY50 were reported to regulate *MdCHS* responsible for increased anthocyanin accumulation under drought stress in apple (Bai et al. 2024). Nevertheless, the contribution and regulation of *CHS* to tangeretin synthesis for cold tolerance is poorly investigated.

Plants have developed a complicated and intertwined signalling transduction network that plays a critical role in the regulation of metabolite accumulation implicated in the adaptation to the constantly changing environment (Ding et al. 2020; Kidokoro et al. 2022). TFs, regarded as molecular switches that integrate extracellular stimuli into transcriptional reprogramming, are indispensable components that function to orchestrate plant responses to both abiotic and biotic stresses through precise regulation of stress‐responsive genes (Liu et al. 2014; Nagano et al. 2019; Kidokoro et al. 2022). Substantial evidence indicates that the tripartite MBW complex, composed of MYBs, basic helix–loop–helix (bHLH) proteins, and WD40 repeat‐containing scaffold proteins, serves as the important regulatory module governing the flavonoid biosynthesis pertinent to plant growth, development and stress adaptation (Feller et al. 2011; Li et al. 2019, 2025; Zhao et al. 2019; Zhou et al. 2019; An et al. 2020). Moreover, increasing data have demonstrated that other TFs, such as WRKY, bZIP and NAC, also regulate flavonoid synthesis through MBW‐dependent or ‐independent pathways (An et al. 2019; Hu et al. 2019; Qiu et al. 2019; Sun et al. 2019). G‐box binding factors (GBFs), a subfamily of plant basic leucine zipper (bZIP) proteins, and nuclear factor Y (NF‐Y) TFs are regarded as critical regulators responsible for various abiotic stress responses (Sun et al. 2015; An et al. 2018; Li et al. 2022; Xie et al. 2024; Yu et al. 2024). However, the mechanistic understanding and associated regulatory network of GBF3 and/or NF‐Y‐mediated flavonoid synthesis and cold tolerance remains to be explored.

Citrus, a globally important fruit crop with high consumer preference and market competitiveness, constitutes a vital agricultural commodity. However, most citrus cultivars exhibit marked cold sensitivity. In our earlier work, metabolomic and transcriptomic profiles of cold‐tolerant Ichang papeda (*Citrus ichangensis*) and cold‐sensitive HB pummelo ( *C. grandis* cv. Hirado Buntan) were illustrated (Xiao, Qu, et al. 2024). Of note, mRNA abundance of *CiCHS2* (Ci190150) and levels of tangeretin, an intermediate of flavonoid synthesis, were remarkably higher in Ichang papeda than in HB pummelo. However, the role of tangeretin in cold tolerance and transcriptional regulation of *CiCHS2* in response to cold stress remains largely elusive. Herein, we first investigated the role of tangeretin and *CiCHS2* in cold tolerance. Next, we identified CiGBF3 and CiNFYA1 as two upstream transcriptional activators of *CiCHS2* by specifically binding to the G‐box (CACGTG) and CCAAT motifs within the promoter. Both CiGBF3 and CiNFYA1 were shown to play a positive role in the regulation of *CiCHS2*‐mediated tangeretin accumulation for rendering cold tolerance. Meanwhile, CiNFYA1 was found to physically associate with CiGBF3 that synergistically activated *CiCHS2* expression under cold stress. In addition, CiNFYA1 was shown to transcriptionally regulate *CiGBF3* by interacting with the gene promoter, further promoting the cold‐responsive upregulation of *CiCHS2*‐mediated tangeretin biosynthesis. Taken together, our findings reveal a regulatory module composed of CiNFYA1‐CiGBF3‐*CiCHS2* in the modulation of tangeretin synthesis for facilitating cold tolerance.

## 2 Results

### 2.1 Elevation of Tangeretin Levels Led to Enhanced Cold Tolerance

**FIGURE 1:**
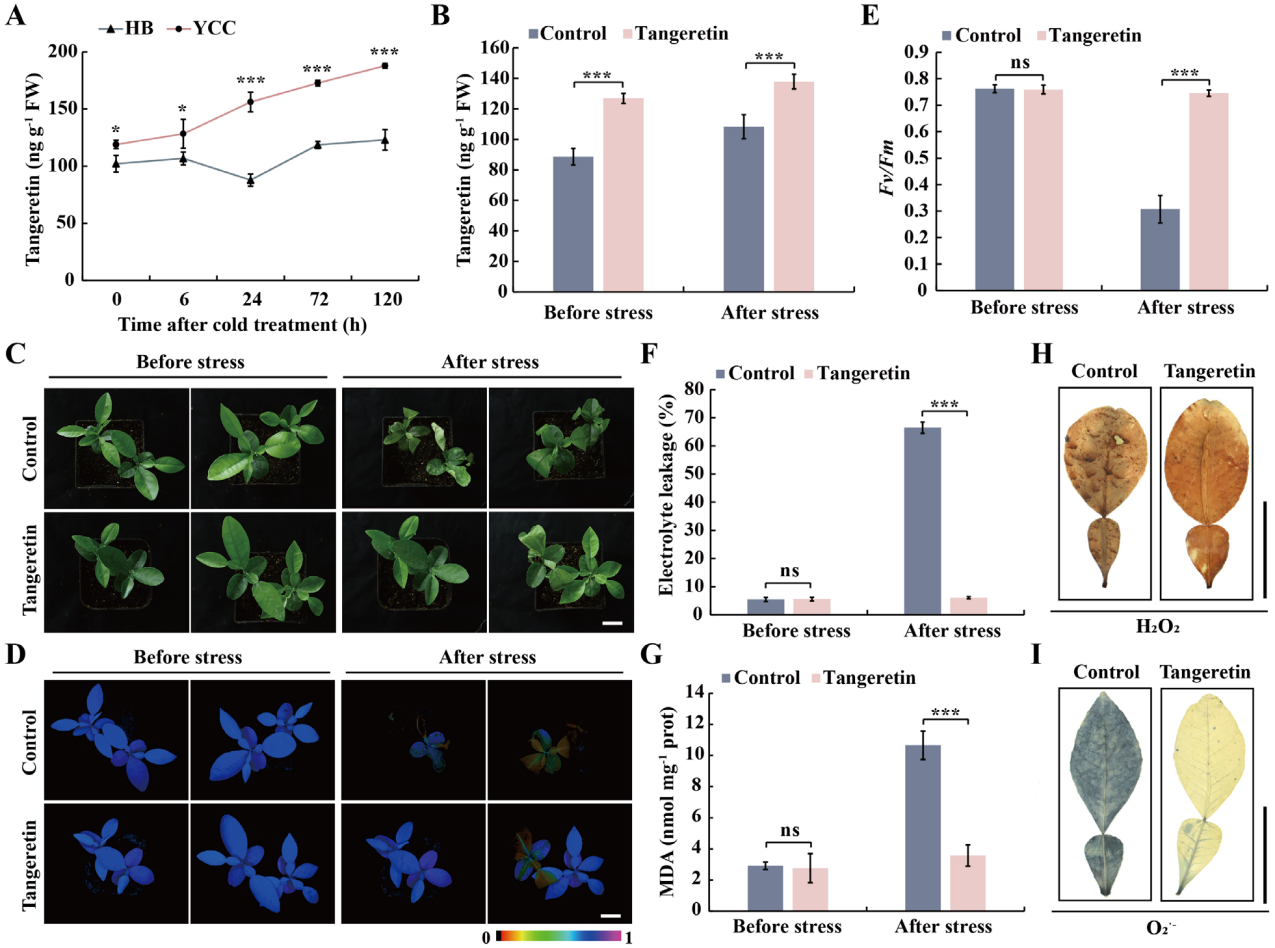
Exogenous tangeretin treatment‐enhanced cold tolerance of HB pummelo. (A) Endogenous tangeretin content in Ichang papeda (Yi Chang Cheng, YCC) and HB pummelo (HB) in response to cold (4°C) treatment. (B) Tangeretin content in HB pummelo seedlings, which had been pretreated with tangeretin (500 μM) or methanol (10%, v/v, Control), before and after cold imposition. (C–E) Phenotypes (C), chlorophyll fluorescence imaging (D) and *Fv/Fm* value (E) of methanol (Control) or tangeretin‐pretreated HB pummelo seedlings, scored before and after cold stress. (F and G) Electrolyte leakage (F) and MDA content (G) of all tested plants before and after cold treatment. (H and I) Histochemical staining with 3,3‐diaminobenzidine (DAB) and nitro blue tetrazolium (NBT) for the detection of in situ accumulation of H_2_O_2_ (H) and O_2_˙^−^ (I), respectively, in the leaves sampled from the HB without or with tangeretin pretreatment. Images in (H) and (I) were digitally extracted for comparison. Scale Bars = 3 cm. FW, fresh weight. Error bars represent ± SD (*n* = 3). Asterisks indicate that the values are significantly different at the same time point or between the involved pairs (based on Student's *t*‐test: *p < 0.05, ***p < 0.001; ns, no significance, *p* > 0.05).

Tangeretin has been shown to exhibit antioxidant properties for improving animal/human health and combating fungus (Liang et al. 2021), but its effect on imparting plant tolerance to abiotic stresses is unknown. To validate cold‐inducible tangeretin accumulation, we examined the tangeretin content in another set of Ichang papeda and HB pummelo plants subjected to cold treatment at 4°C. Consistent with the metabolomic datasets (Figure S1), the endogenous tangeretin of Ichang papeda was significantly and steadily induced over a 120‐h period, whereas the tangeretin levels in HB pummelo only exhibited a slight elevation (Figure 1A). As a result, the tangeretin levels in the cold‐tolerant genotype were substantially higher than those in the cold‐sensitive one in the presence of cold treatment, and the difference was particularly significant at the later stages. Therefore, we were curious to know whether tangeretin plays a role in modulation of cold tolerance. To answer this question, we first performed the cold tolerance assays of citrus plants treated with or without exogenous tangeretin. To this end, we first conducted preliminary experiments by comparing cold tolerance of citrus shoots treated with tangeretin using five concentrations: 0, 100, 250, 500, 800 μM. The results showed that cold tolerance was enhanced by tangeretin in a concentration‐dependent manner below 500 μM, whereas the stimulatory effect was stable when the concentration was increased to 800 μM (Figure S2). Therefore, we selected 500 μM as an optimum concentration for this work. Exogenous tangeretin supply significantly increased tangeretin content relative to the control (Figure 1B). In comparison with the seedlings without supply of tangeretin, the application of exogenous tangeretin substantially enhanced the cold tolerance, as manifested by a reduction in leaf wilting under cold exposure (Figure 1C). In accordance with the phenotype, stronger chlorophyll fluorescence and higher *Fv/Fm* ratio, but lower electrolyte leakage (EL) and malondialdehyde (MDA) levels, were observed in the tangeretin‐treated plants (Figure 1D–G). Meanwhile, the leaves of tangeretin‐pretreated plants accumulated prominently less H_2_O_2_ and O_2_˙^−^, as revealed by 3,3‐diaminobenzidine (DAB) and nitro blue tetrazolium (NBT) staining. Furthermore, exogenous tangeretin at 500 μM was sprayed on lemon ( *Citrus limon* ), another cold‐sensitive citrus, prior to cold treatment. Likewise, we found that exogenous application of tangeretin also resulted in significant elevation of cold resistance of lemon (Figure S3). These results demonstrate that exogenous tangeretin supplementation led to enhanced cold tolerance.

### 2.2 CiCHS2 Was Induced by Cold and Localised in the Endoplasmic Reticulum (ER)

**FIGURE 2:**
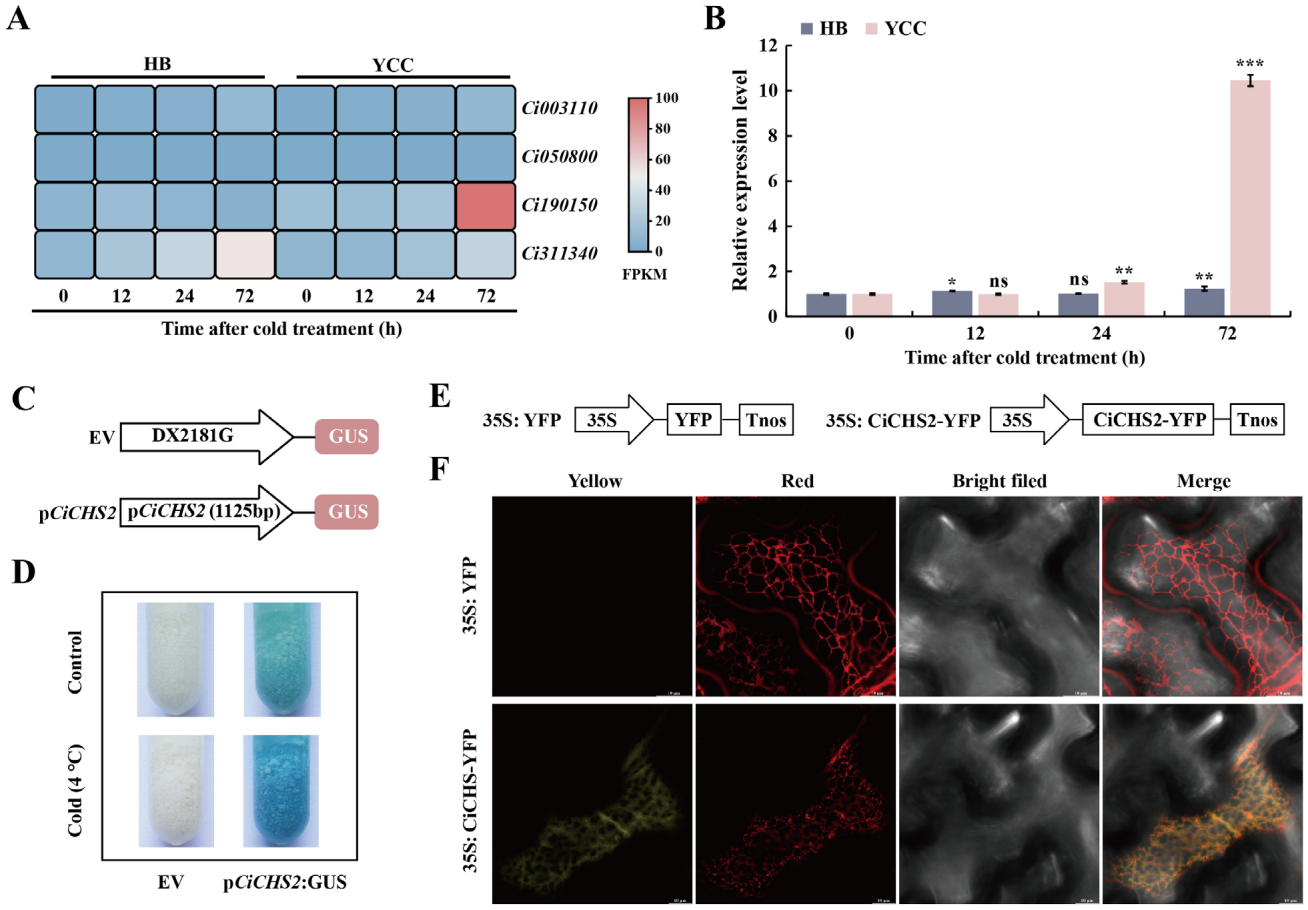
Cold‐induced *CiCHS2* expression and analysis of CiCHS2 localisation. (A) Fragments Per Kilobase of exon per Million (FPKM) values of *CHS* genes in Ichang papeda (Yi Chang Cheng, YCC) and HB pummelo (HB) based on RNA‐seq datasets. (B) Comparison of expression patterns of *CiCHS2* (*Ci90150*) between Ichang papeda and its homologue in HB pummelo under cold treatment, as analysed by RT‐qPCR. (C, D) Schematic diagrams (C) and GUS staining (D) of sweet orange ( *C. sinensis* ) callus transformed with the empty vector DX2181G (EV) or p*CiCHS2*: GUS construct (p*CiCHS2*) with or without cold treatment. (E) Schematic diagrams of YFP (yellow fluorescent protein) empty vector (35S: YFP) or fusion construct (35S: CiCHS2‐YFP) used for the subcellular localisation. (F) Confocal microscopic images of tobacco (*Nicotiana benthamiana*) epidermal cells cotransformed with 35S: YFP or 35S: CiCHS2‐YFP, along with an endoplasmic reticulum (ER) marker gene HDEL fused to OFP (orange fluorescent protein, 35S: OFP‐HDEL). Scale bars = 10 μm. Data are means ± SD (*n* = 3). Asterisks indicate that the values are significantly different that of 0 h (based on Student's *t*‐test: *p < 0.05, **p < 0.01, ***p < 0.001; ns, no significance, *p* > 0.05).

There are four genes annotated as chalcone synthase (CHS) in the transcriptome dataset. Analysis of the expression of these CHS genes showed that Ci190150 (*CiCHS2*) of Ichang papeda exhibited the greatest induction by cold treatment (Figure 2A). This result was further confirmed by RT‐qPCR analysis (Figure 2B). In order to verify the cold‐inducible feature of *CiCHS2*, we generated transgenic embryogenic callus of sweet orange transiently expressing *β*‐glucuronidase (GUS) reporter gene driven by a 1125‐bp promoter of *CiCHS2* (p*CiCHS2*) (Figure 2C). Histochemical staining showed that the GUS activity was detected in the p*CiCHS2*: GUS‐expressing callus under normal growth conditions and prominently activated following the cold exposure (Figure 2D). Collectively, these findings indicate that *CiCHS2* was induced in a substantial manner by cold exposure. In addition, to gain a deeper understanding of its function, subcellular localisation of CiCHS2 was examined, which showed that the YFP signal of 35S: CiCHS2‐YFP was completely overlapping with that of the ER marker protein HDEL (Figure 2E,F), implying that CiCHS2 was localised in the ER.

### 2.3 *CiCHS2* Contributed to Cold Tolerance via Modulating Tangeretin Biosynthesis

**FIGURE 3:**
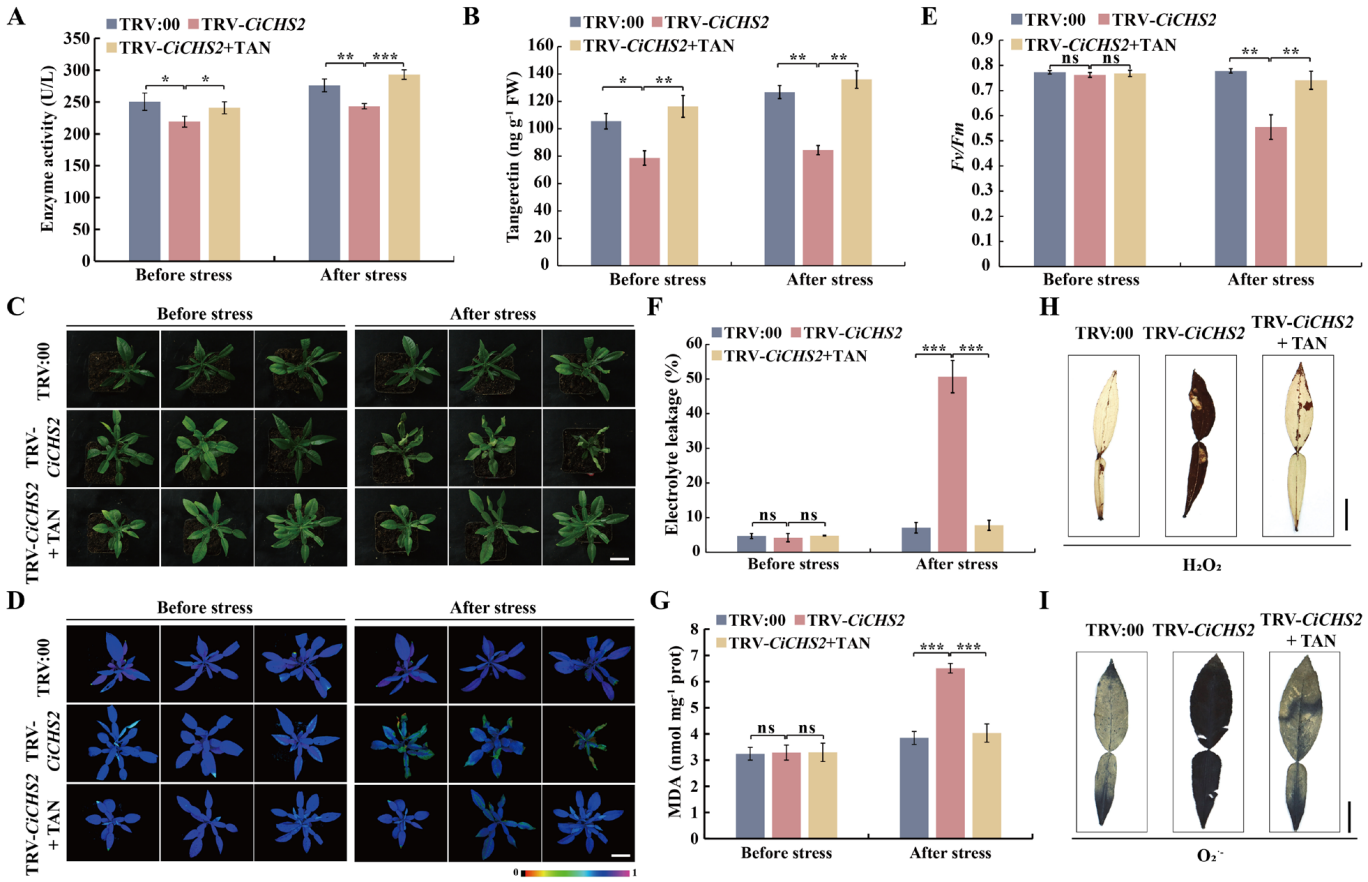
Silencing of *CiCHS2* by virus‐induced gene silencing (VIGS) decreased cold tolerance in Ichang papeda. (A, B) Chalcone synthase activity (A) and tangeretin contents (B) measured before and after cold treatment of the TRV:00 plants and the VIGS plants that had been pretreated without or with 500 μM tangeretin (TAN). C‐G, Phenotypes (C), chlorophyll fluorescence imaging (D), *Fv/Fm* ratios (E) electrolyte leakage (F), and MDA content (G) in the TRV:00 and VIGS plants, mentioned in (A). The false colour scale is shown below the imaging. (H, I) Histochemical staining with 3,3‐diaminobenzidine (DAB) and nitro blue tetrazolium (NBT) for detection of in situ accumulation of H_2_O_2_ (H) and O_2_˙^−^ (I), respectively, in the leaves sampled from the cold‐treated TRV:00 control and TRV‐*CiCHS2* plants without or with tangeretin pretreatment. Images in (H) and (I) were digitally extracted for comparison. Scale bars, 3 cm (C and D) or 2 cm (H, I). FW, fresh weight. Error bars indicate ± SD (*n* = 3). Asterisks indicate that the values are significantly different between the involved pairs (based on Student's *t*‐test: *p < 0.05, **p < 0.01, ***p < 0.001; ns, no significance, *p* > 0.05).

The fact that *CiCHS2* was induced by cold suggests that it may play a role in the modulation of cold tolerance. To verify this assumption, we first employed the tobacco rattle virus (TRV)‐mediated virus‐induced gene silencing (VIGS) approach to knock down *CiCHS2* in *C. ichangensis*. *CiCHS2* transcript levels were significantly reduced in the VIGS plants (TRV‐*CiCHS2*) compared with the TRV:00 control (Figure S4). The VIGS plants (TRV‐*CiCHS2*) exhibited reduced enzyme activity and tangeretin level compared with the TRV:00 control before and under the cold stress (Figure 3A,B). Under normal growth conditions, no observable phenotypic or physiological difference was noted between the VIGS and the control plants. Upon cold exposure, the VIGS plants exhibited more serious leaf damage, which was further corroborated by weaker chlorophyll fluorescence and lower *Fv/Fm* ratio, in comparison with the TRV:00 plants (Figure 3C–E). In addition, the cellular damage was more evident in the TRV‐*CiCHS2* plants compared with the control line, as reflected by higher EL and MDA levels (Figure 3F,G). Meanwhile, the DAB and NBT revealed a greater degree of staining in the leaves of TRV‐*CiCHS2* plants compared with the control in the presence of cold treatment, implying that endogenous H_2_O_2_ and O_2_˙^−^ levels were noticeably increased (Figure 3H,I). Interestingly, the application of exogenous tangeretin to the TRV‐*CiCHS2* plants prior to the cold treatment resulted in an appreciable resumption of the cold‐sensitive phenotype observed in the VIGS line, which was substantiated through the evaluation of the aforementioned parameters.

**FIGURE 4:**
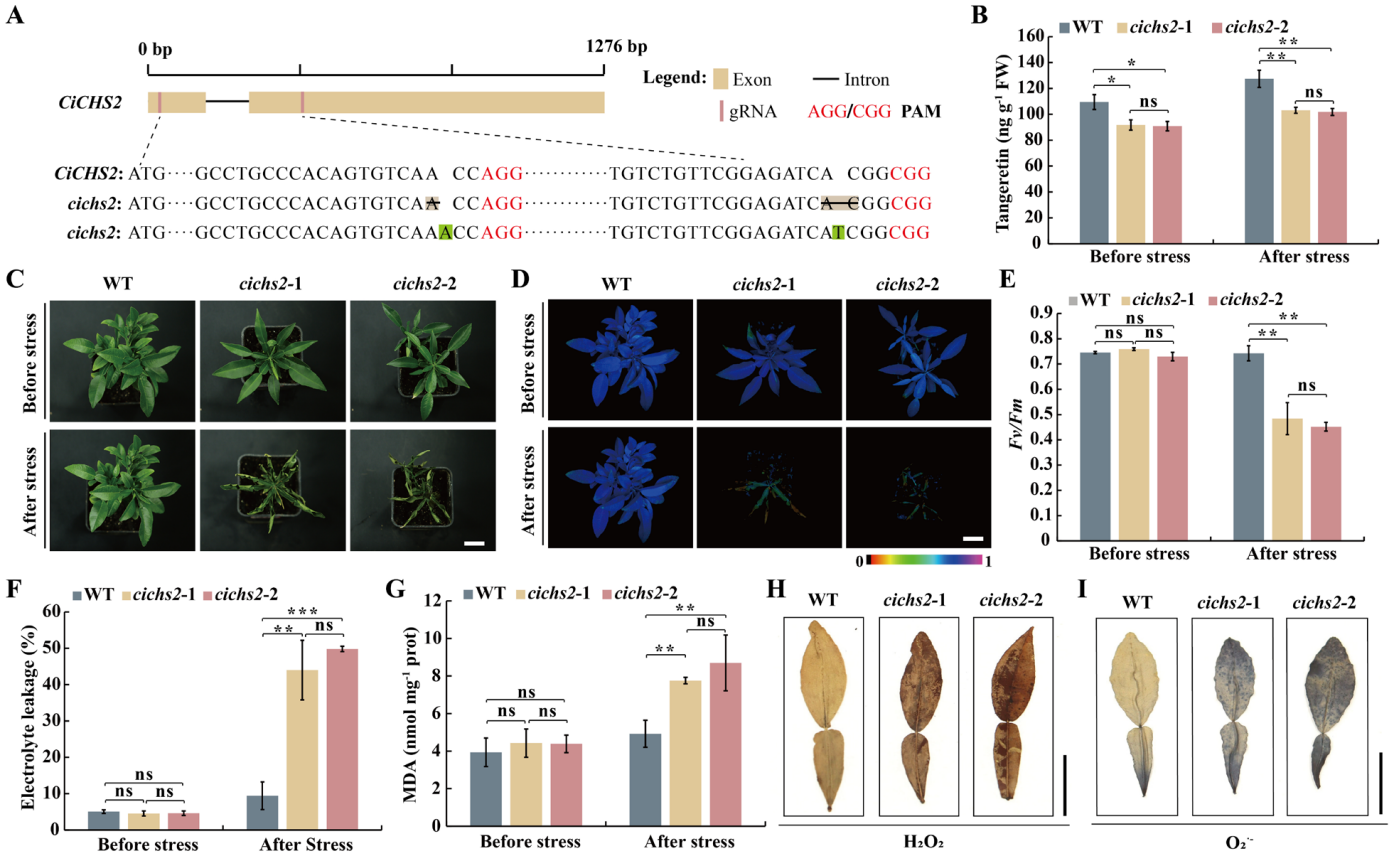
CRISPR‐Cas9‐based *cichs2* mutants exhibited cold sensitivity. (A) gRNA sequences of wild‐type (WT) and the gene‐edited mutant lines. The protospacer adjacent motif (PAM) of two gRNA were marked in red, while base deletion and insertion were shaded in orange and green, respectively. (B) Tangeretin content in the WT and mutant lines before and after the cold treatment. (C–G) Phenotypes (C), chlorophyll fluorescence imaging (D) and *Fv/Fm* ratios (E), electrolyte leakage (F), and MDA content (G) of WT and mutants before and after the cold treatment. (H, I) Histochemical staining with 3,3‐diaminobenzidine (DAB) and nitro blue tetrazolium (NBT) for the detection of in situ accumulation of H_2_O_2_ (H) and O_2_˙^−^ (I), respectively, in the leaves sampled from the WT and *cichs2* mutants. Scale bars, 3 cm (C, D) or 2 cm (H, I). Images in (H) and (I) were digitally extracted for comparison. FW, fresh weight. Error bars indicate ± SD (*n* = 3). Asterisks indicate that the values are significantly different between the involved pairs (based on Student's *t*‐test: *p < 0.05, **p < 0.01, ***p < 0.001; ns, no significance, *p* > 0.05).

To confirm the results of VIGS, two *cichs2* mutant lines (*cichs2*‐1 *and cichs2*‐2) were generated using CRISPR/Cas9‐based genome editing of Ichang papeda. Deletion of one and two bases was observed in the two guide RNA for *cichs2*‐1, whereas *cichs2*‐2 contained an insertion in each (Figure 4A). In comparison with WT, the *cichs2* mutants showed significantly reduced tangeretin content, particularly under cold stress (Figure 4B). The mutant buds were grafted onto Ichang papeda so as to get complete plants in the short term. The mutation of *CiCHS2* did not affect the leaf shape under standard growth conditions. However, the edited lines exhibited manifested and more severe cold damage under cold stress relative to the WT, as evidenced by the difference in degree and severity of leaf wilting (Figure 4C). In the presence of cold treatment, the two *cichs2* mutants exhibited weaker chlorophyll fluorescence, lower *Fv/Fm* ratio, but higher EL and MDA levels, compared with the WT, although these parameters were equivalent between the examined genotypes under normal growth conditions (Figure 4D–G). Moreover, the levels of H_2_O_2_ and O_2_˙^−^ were profoundly higher in the *cichs2* mutants than in the WT, as revealed by DAB and NBT staining (Figure 4H,I).

**FIGURE 5:**
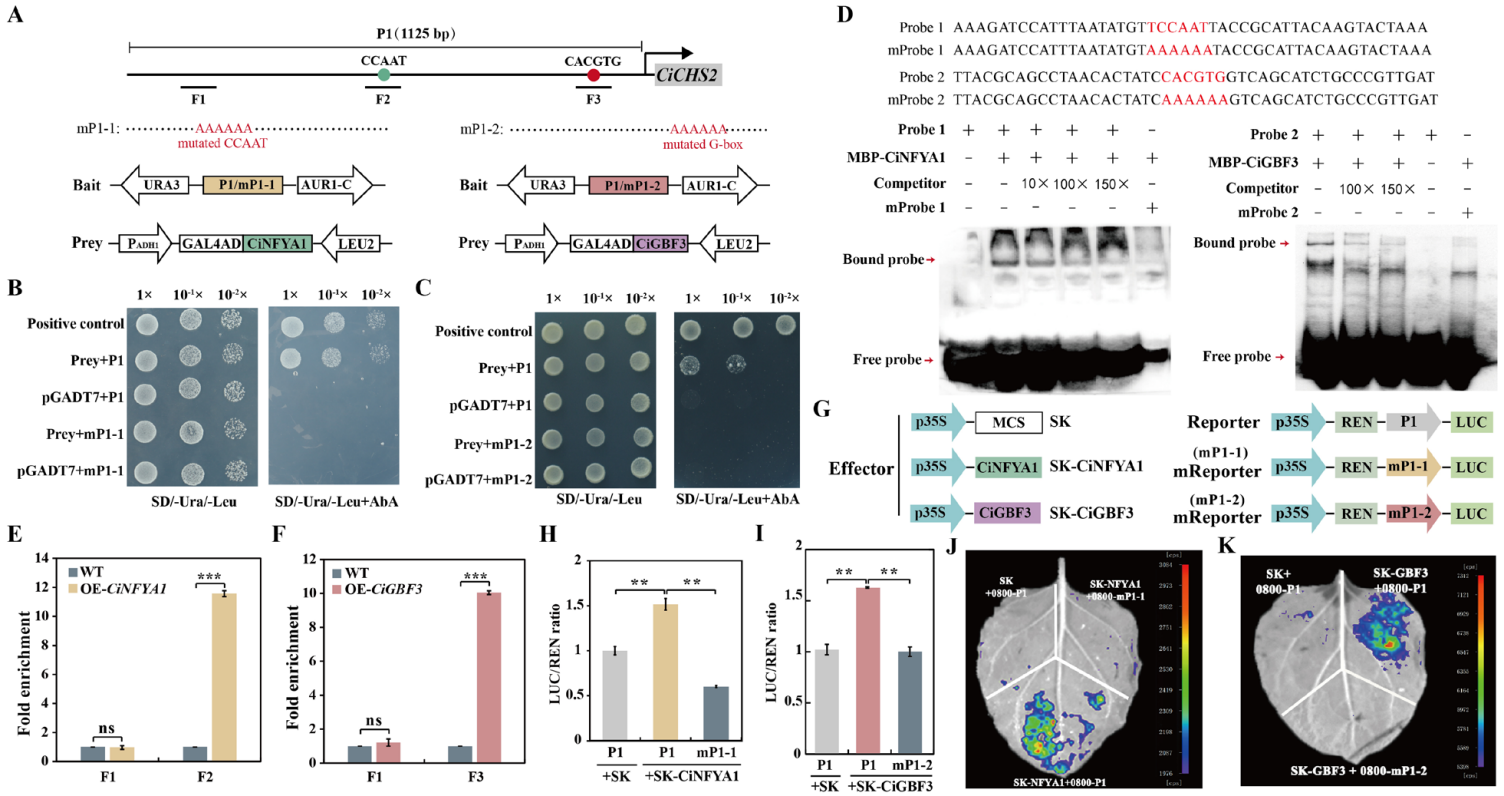
CiGBF3 and CiNFYA1 bind to and activate the promoter of *CiCHS2*. (A) Schematic diagram of the *CiCHS2* promoter (p*CiCHS2*) and distribution of *cis*‐acting elements. P1 and mP1‐1/2 are the promoter fragments with original or mutated G‐box and CCAAT motifs. F1, F2 and F3 are three fragments used for ChIP assay. (B and C) Growth of yeast cells co‐transformed with pGADT7‐ CiNFYA1 (B) or pGADT7‐ CiGBF3 (C) and the corresponding baits on Synthetic Dropout (SD)/–Ura/−Leu selective medium without or with AbA. p53‐AbAi + pGADT7‐p53 and bait + pGADT7 were used as positive and negative control, respectively. (D) EMSA analysis of interaction between CiNFYA1 or CiGBF3 and p*CiCHS2*. The His‐ CiNFYA1 or His‐ CiGBF3 fusion protein was incubated with the biotin‐labelled probe containing the CCAAT (Probe 1) and G‐box (Probe 2) or their mutated counterparts (mProbe1 for CCAAT, mProbe2 for G‐box). + and −represent the presence or absence of the components shown on the top. E‐F, ChIP‐qPCR using primers specific to F1, F2 and F3 for revealing the enrichment of CiNFYA1 (E) or CiGBF3 (F) in p*CiCHS2*. (G) Schematic diagrams of the effector and reporter vectors used for the dual luciferase (LUC) assays. p35S, the CaMV 35S promoter; LUC, firefly luciferase; MCS, multiple cloning sites; REN, *Renilla* luciferase. (H–K) LUC/REN ratios (H, I) and LUC bioluminescence imaging (J, K) in *N. benthamiana* leaves transiently expressing SK‐CiNFYA1 (H, J), SK‐CiGBF3 (I, K), along with SK, with the reporters containing original or mutated motifs. SK + p*CiCHS2* was used as a negative control, in which the LUC/REN ratio was set to 1 for normalisation. Error bars indicate ± SD (*n* = 3). Asterisks indicate that the values are significantly different between the involved pairs (based on Student's *t*‐test; **p < 0.01, ***p < 0.001; ns, no significance, *p* > 0.05).

Moreover, we ectopically overexpressed *CiCHS2* in tobacco to generate transgenic plants, from which two independent lines (Figure S5), #3 and #9, were selected for cold tolerance assay. Under normal growth conditions, no morphological difference was observed between 4‐week‐old transgenic and WT tobacco plants. However, the transgenic plants displayed obviously better growth phenotype, as reflected by less serious leaf damage in comparison with the WT when subjected to cold stress (Figure S6A). Consistent with the phenotypic observation, the transgenic lines displayed stronger chlorophyll fluorescence and higher *Fv/Fm* ratio, together with lower EL and MDA levels, compared with the WT in the presence of cold treatment (Figure S6B–E). In addition, the transgenic plants had higher CHS activity and tangeretin levels relative to the WT without or with cold stress (Figure S6F,G). Following the cold treatment, the leaves of transgenic plants accumulated prominently less H_2_O_2_ and O_2_˙^−^ relative to the WT (Figure 5H). Furthermore, a similar phenotype was observed when 7‐week‐old tobacco plants were subjected to cold treatment (Figure S6I). Taken together, these data show that the overexpression of *CiCHS2* led to enhanced cold tolerance in the transgenic plants. Collectively, the results mentioned above indicate that *CiCHS2* plays a positive role in the modulation of cold tolerance by promoting tangeretin biosynthesis.

### 2.4 CiGBF3 and CiNFYA1 Directly Activated *CiCHS2* Expression

To identify the transcriptional regulators of *CiCHS2*, its promoter (p*CiCHS2*, 1125 bp) was used to construct a yeast one‐hybrid (Y1H) bait for screening a cDNA library derived from *C. ichangensis*. Among the sequenced clones (Table S1, Figure S7), two candidates annotated as nuclear transcription factor Y subunit A (NFYA) and basic leucine zipper (bZIP) protein (Figure S8) were selected for further analysis, as p*CiCHS2* contained the CCAAT (CCAAT, −564 to −568 bp) and G‐box (CACGTG, −134 bp to −139 bp) motifs. Phylogenetic analysis showed that they were most closely related to NFYA1 and GBF3 of *A. thaliana* ; respectively, they were thus named as CiNFYA1 and CiGBF3 (Figures S9 and S10). Furthermore, both CiNFYA1 and CiGBF3 were shown to be nuclear proteins (Figure S11A,B). In addition, transcription activation activity assays showed that CiNFYA1 and CiGBF3 could activate the luciferase (LUC) reporter gene, suggesting that CiNFYA1 and CiGBF3 possess transcription activation activity (Figure S11C–G).

A point‐to‐point Y1H assay was first performed to verify the interaction between CiNFYA1 or CiGBF3 and the p*CiCHS2*‐derived bait (Figure 5A). Consistent with the positive control, the yeast cells cotransformed with the preys and the p*CiCHS2* bait exhibited normal growth on the selection medium supplemented with Aureobasidin A (AbA). By contrast, mutation of either the CCAAT motif (TCCAAT to AAAAAA) or the G‐box (CACGTG to AAAAAA) in p*CiCHS2* completely inhibited the yeast growth, implying that CiNFYA1 and CiGBF3 could bind to p*CiCHS2* via the two *cis*‐acting elements (Figure 5B,C). Subsequently, an electrophoretic mobility shift assay (EMSA) was carried out to verify the in vitro interaction. Incubation of His‐CiNFYA1 and a probe containing the CCAAT motif or incubation of His‐CiGBF3 and a probe harbouring the G‐box results in an obvious band shift. However, the band migration was impaired by adding the unlabelled competitor DNA in a dosage‐dependent manner, whereas the band shift was completely abolished when the CCAAT or G‐box sequence was mutated (Figure 5D). Moreover, chromatin immunoprecipitation (ChIP)‐qPCR assay with GFP antibody using transgenic citrus calli expressing 35S::CiNFYA1‐GFP or 35S::CiGBF3‐GFP was performed. The promoter regions containing the CCAAT and G‐box motifs were prominently enriched, while the regions without the two elements showed no enrichment (Figure 5E,F). These results confirm that CiNFYA1 and CiGBF3 directly and specifically bound to the CCAAT and G‐box elements of p*CiCHS2*, respectively.

To further validate the transcriptional activation pattern of CiNFYA1 and CiGBF3, we performed a dual luciferase (LUC)‐based transient examination. The effectors (SK‐CiNFYA1 or SK‐CiGBF3) and the reporters driven by p*CiCHS2* (Figure 5G) were subsequently coinfiltrated in the leaves of *Nicotiana benthamiana*. Coexpression of each effector and the reporter led to significant elevation of the LUC activity compared with the control, in which the empty effector vector was coinfiltrated with the reporter. However, when the G‐box and CCAAT motifs were mutated, the LUC activity was prominently inhibited (Figure 5H,I). The quantitative assays were further supported by LUC fluorescence imaging, as reflected by the increased LUC expression when the reporter was coexpressed with SK‐CiNFYA1 or SK‐CiGBF3 in comparison with the control (Figure 5J,K). Taken together, these results indicate that CiGBF3 and CiNFYA1 functioned as transcriptional activators of *CiCHS2*.

### 2.5 CiGBF3 and CiNFYA1 Function Positively in Cold Tolerance by Modulating *CiCHS2* ‐Mediated Tangeretin Synthesis

**FIGURE 6:**
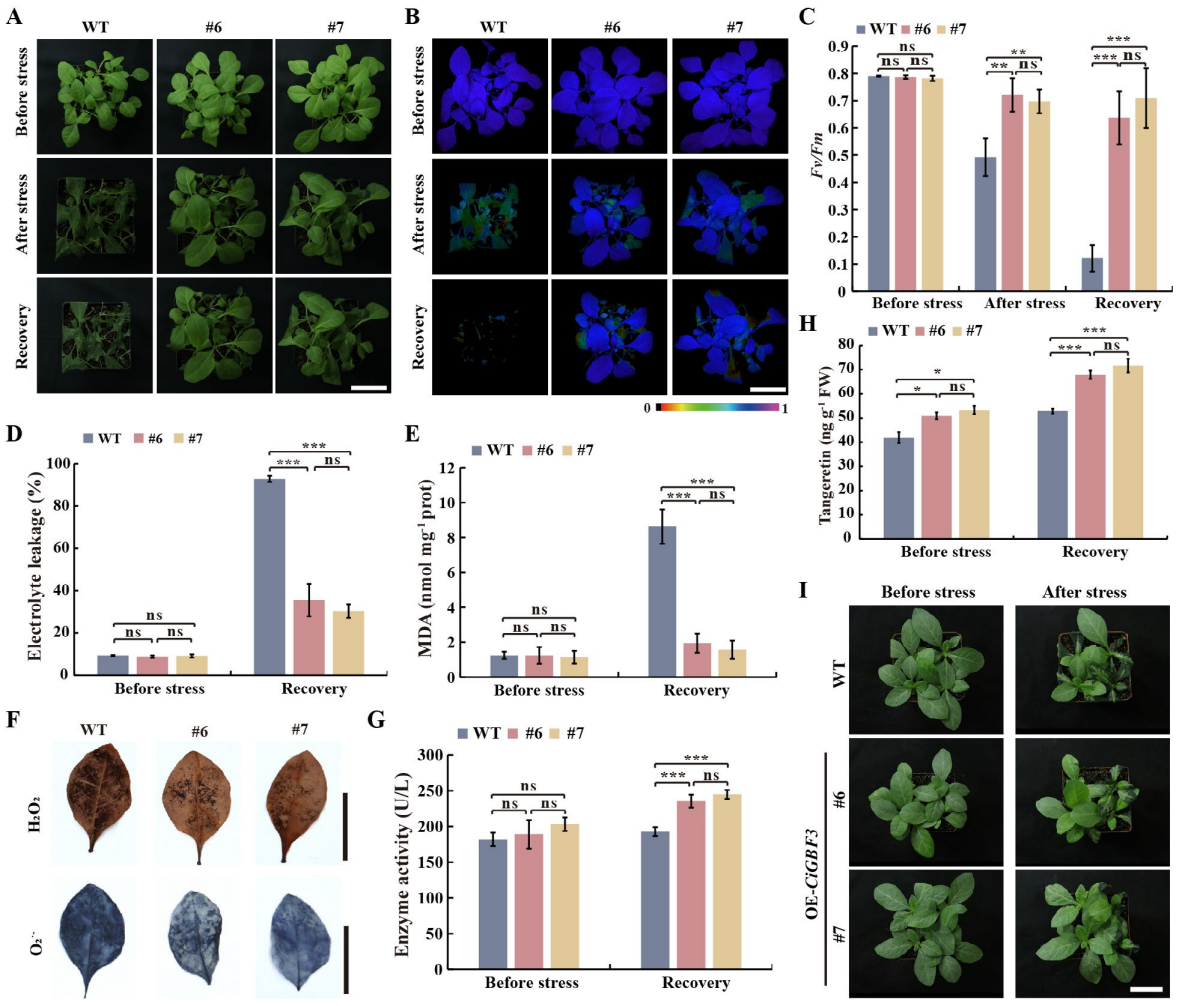
Overexpression of *CiGBF3* improved cold tolerance in transgenic tobacco. (A–C) Phenotypes (A), chlorophyll fluorescence (B) and *Fv/Fm* ratios (C) of 4‐week‐old overexpression lines (#6 and #7) and wild‐type (WT), scored before, after the cold treatment, and after the growth recovery for 1 day at 25°C. The false colour scale is shown below the imaging. (D, E) Electrolyte leakage (D) and MDA contents (E) in the tested plants measured before and after the growth recovery. (F) In situ accumulation of H_2_O_2_ (upper panel) and O_2_˙^−^ (bottom panel) in the transgenic plants and WT after cold treatment, as detected by histochemical staining with 3,3‐diaminobenzidine (DAB, upper panel) and nitro blue tetrazolium (NBT, bottom panel). (G and H) CHS activity (G) and tangeretin content (H) in the plants before and after the cold treatment. (I) Phenotypes of 7‐week‐old transgenic and WT plants after the cold treatment. Scale bars = 4 cm. FW, fresh weight. Error bars indicate ± SD (*n* = 3). Asterisks indicate that the values are significantly different between the involved pairs (based on Student's *t*‐test; *p < 0.05, **p < 0.01, ***p < 0.001; ns, no significance, *p* > 0.05).

Transcript levels of *CiGBF3* were progressively increased to reach the peak at 72 h in Ichang papeda under cold treatment, implying that *CiGBF3* may play a role in modulation of cold tolerance (Figure S8). To verify this assumption, *CiGBF3*‐overexpressing (OE) tobacco transgenic plants were generated, from which two OE lines (#6 and #7) were selected for further cold tolerance assessment (Figures S12 and S13). No phenotypic variations were noticed between the WT and OE lines under normal growth conditions. Upon exposure to cold treatment (2°C for 2 h prior to −2°C for 5 h) and growth recovery, the WT plants exhibited more serious leaf withering and plant growth repression in comparison with the OE plants (Figure 6A). In line with the morphological change, the OE plants had obviously stronger chlorophyll fluorescence and higher *Fv/Fm* ratios, accompanied by lower levels of EL and MDA and less ROS, relative to the WT after the cold treatment (Figure 6B–F). In addition, the CHS activity and tangeretin levels in the OE lines were higher, slightly before but significantly after the cold treatment, than those in the WT to the WT (Figure 6G,H). Likewise, when 7‐week‐old tobacco plants were subjected to cold treatment, the WT exhibited more pronounced growth damage when compared to the transgenic lines (Figure 6I). These results indicate that heterologous expression of *CiGBF3* conferred enhanced cold tolerance.

**FIGURE 7:**
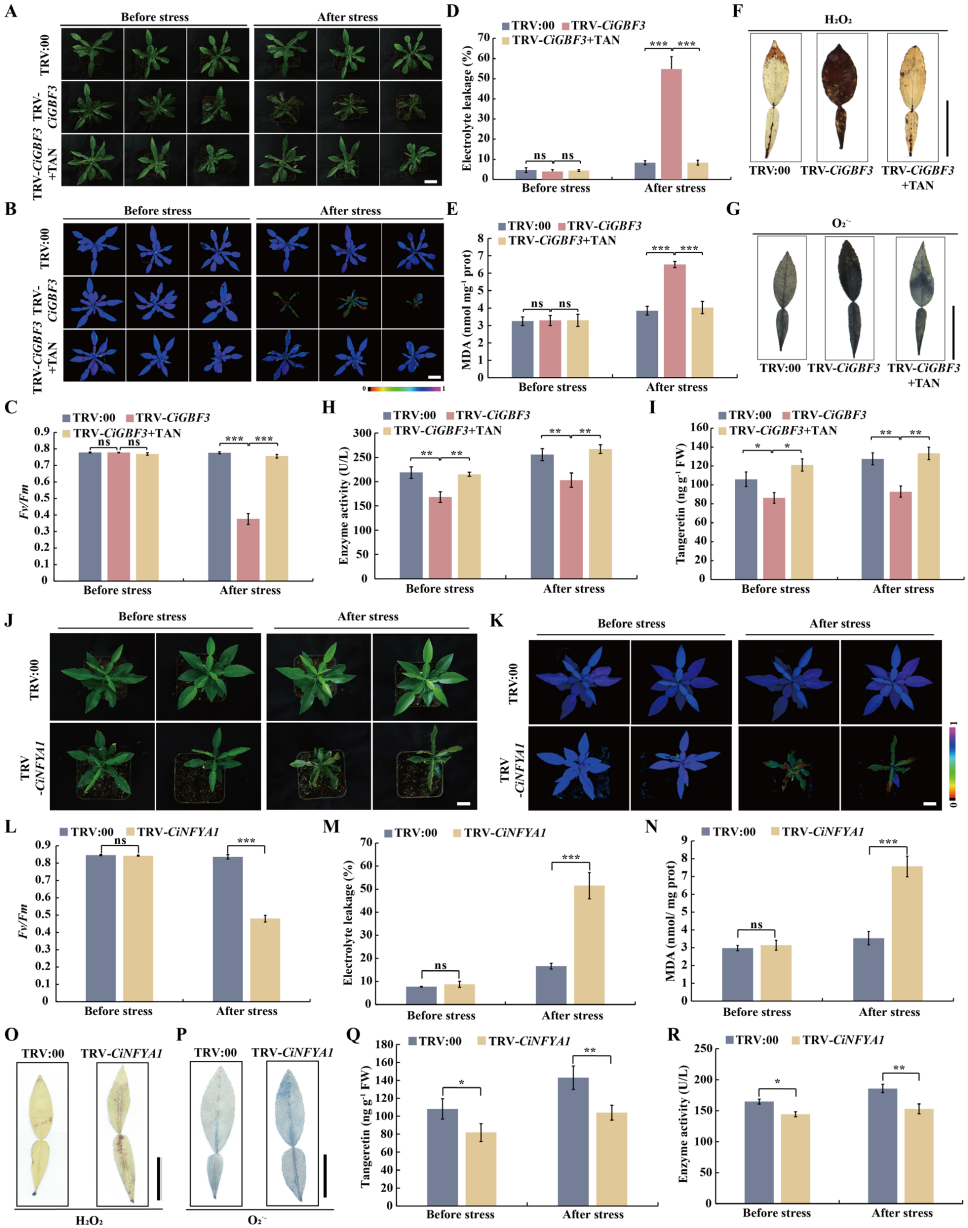
Knockdown of *CiGBF3* or *CiNFYA1* in Ichang papeda impaired cold tolerance. (A–C) Phenotypes (A), chlorophyll fluorescence imaging (B) and *Fv/Fm* ratios (C) examined before and after cold treatment of the TRV:00 control, VIGS plants (TRV‐*CiGBF3*) pretreated with or without exogenous 500 μM tangeretin (TAN). (D, E) Electrolyte leakage (D) and malondialdehyde (MDA) content (E) detected before and after cold treatment in the cold‐treated TRV:00 control, TRV‐*CiGBF3* plants with or without tangeretin pretreatment. (F, G) In situ accumulation of H_2_O_2_ (F) and O_2_˙^−^ (G) in the leaves detected after the cold treatment of the tested plants, as revealed by histochemical staining with 3,3‐diaminobenzidine (DAB) and nitro blue tetrazolium (NBT), respectively. (H, I) CHS activity (H) and tangeretin content (I) detected before and after cold treatment of all tested plants. (J–N) Phenotypes (J), Chlorophyll fluorescence imaging (K), *Fv/Fm* ratios (L), electrolyte leakage (M) and MDA content (N) of VIGS (TRV2‐*CiNFYA1*) and TRV:00 control before and after the cold treatment. (O, P) in situ accumulation of H_2_O_2_ (O) and O_2_˙^−^ (P) in the leaves of the tested plants after the cold treatment, as revealed by histochemical staining with 3,3‐diaminobenzidine (DAB) and nitro blue tetrazolium (NBT), respectively. (Q, R) Endogenous tangeretin content (Q) and CHS activity (R) in the VIGS (TRV2‐*CiNFYA1*) and TRV:00 control before and after the cold treatment. Scale bars, 3 cm (A, B and F, G) or 2 cm in (J, K and O, P). FW, fresh weight. Error bars indicate ± SD (*n* = 3). Asterisks indicate that the values are significantly different between the involved pairs (based on Student's *t*‐test; *p < 0.05, **p < 0.01, ***p < 0.001; ns, no significance, *p* > 0.05).

To further explore the biological roles of *CiGBF3* in modulating cold tolerance and tangeretin biosynthesis, *CiGBF3* in *C. ichangensis* was silenced by VIGS. In the VIGS lines, the expression levels of *CiGBF3* and *CiCHS2* were significantly repressed (Figures S14 and S15). Upon exposure to the cold treatment and growth recovery, the VIGS lines exhibited obviously impaired cold tolerance, as reflected by more serious leaf curling and wilting, in comparison with the TRV:00 control plants (Figure 7A). Consistent with the plant morphology, the VIGS line displayed a significant reduction of chlorophyll fluorescence intensity and *Fv/Fm* ratios, along with increased levels of EL, MDA, and ROS, when compared to the TRV:00 control (Figure 7B–G). CHS activity and tangeretin levels were significantly decreased in the TRV‐*CiGBF3* plants compared with the control plants (Figure 7H,I). Interestingly, exogenous tangeretin application to the VIGS plants considerably restored their freezing tolerance phenotype compared with VIGS plants without the tangeretin supply, which was supported by examination of the parameters mentioned above (Figure 7A–I). These data indicate that knockdown of *CiGBF3* substantially reduced tangeretin accumulation and compromised cold tolerance, implying that *CiGBF3* functions positively in cold tolerance through regulating tangeretin synthesis.

**FIGURE 8:**
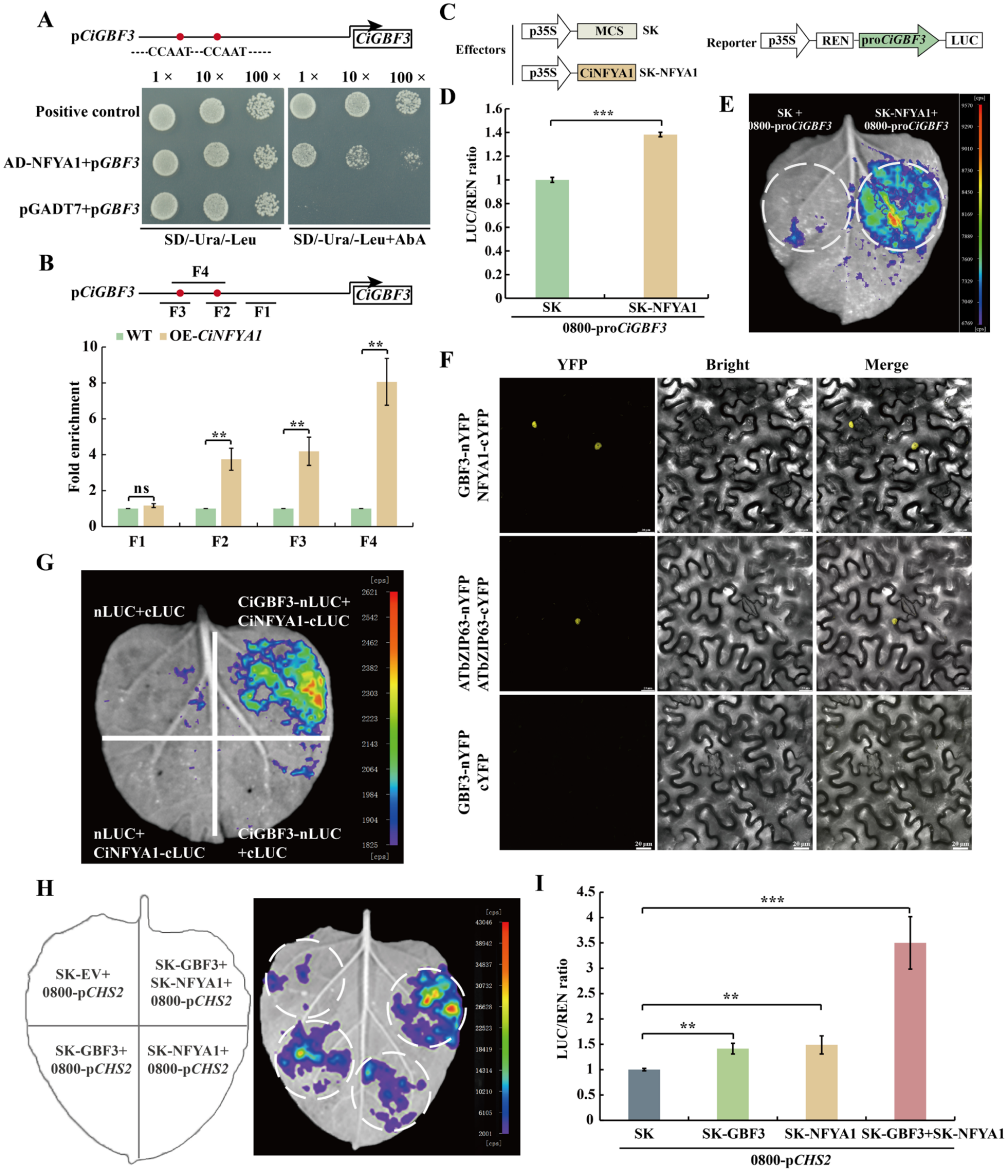
CiNFYA1 transcriptionally activated and physically interacted with CiGBF3. (A) Growth of yeast cells transformed with the prey (pGADT7‐CiNFYA1) and baits constructed using the promoter of *CiGBF3* on synthetic dropout (SD)/–Ura/−Leu selective medium added without or with AbA. p53‐AbAi + pGADT7‐p53 and bait + pGADT7 were used as positive and negative control, respectively. p*CiGBF3*, the promoter of *CiGBF3* with two CCAAT motifs, which are shown in red circles. (B) ChIP‐qPCR assays revealing enrichment of CiNFYA1 in p*CiGBF3* using primers specific to F1–F4. (C) Schematic diagrams of the vectors used for the dual luciferase (LUC) assays. p35S, the CaMV 35S promoter; LUC, firefly luciferase; MCS, multiple cloning sites; REN, *Renilla* luciferase. (D, E) LUC/REN ratios (D) and LUC bioluminescence imaging (E) in *N. benthamiana* leaves transiently expressing the SK‐CiNFYA1 with reporters containing p*CiGBF3*. SK + p*CiGBF3* was used as a negative control, in which the LUC/REN ratio was set to 1 for normalisation. (F) BiFC assay for verifying the interaction between CiNFYA1 and CiGBF3. Yellow fluorescence protein (YFP) signals of *N. benthamiana* leaves infiltrated with GBF3‐nYFP+NFYA1‐cYFP, ATbZIP63 ( *A. thaliana* bZIP63)‐nYFP+ATbZIP63‐cYFP (positive control), and GBF3‐nYFP+cYFP (negative control), were detected under 514‐nm laser light or bright field. (G) LCI assay revealing the in vivo interaction between CiNFYA1 and CiGBF3 by observing the LUC fluorescence in the *N. benthamian* leaves coinfiltrated with the constructs. (H, I) LUC/REN ratios (H) and LUC bioluminescence imaging (I) in *N. benthamiana* leaves cotransformed with the reporter driven by p*CiCHS2* (0800‐p*CiCHS2*) and the effectors, SK‐CiGBF3 and SK‐CiNFYA1, alone or together. SK + p*CiCHS2* was used as a negative control. Error bars indicate ± SD (*n* = 3). Asterisks indicate that the values are significantly different between the involved pairs (based on the Student's *t*‐test; **p < 0.01, ***p < 0.001; ns, no significance, *p* > 0.05).

We also investigated the functions of *CiNFYA1* in regulating cold tolerance by silencing *CiNFYA1* in Ichang papeda via VIGS. The transcript levels of *CiNFYA1* and *CiCHS2* were tremendously reduced in the VIGS plants relative to the TRV:00 control (Figures S16 and S17). Under normal growth conditions, both the VIGS lines and the TRV:00 control plants exhibited consistent growth. However, the TRV‐*CiNFYA1* plants exhibited more severe growth inhibition, as manifested by leaf curling and wilting, in comparison with the control plants under cold treatment (Figure 8J). In agreement with the phenotype, the VIGS plants exhibited significantly weaker chlorophyll fluorescence and lower *Fv/Fm* ratio, but higher levels of EL, MDA and ROS, compared with the control plants in the presence of cold treatment, although these indicators were comparable across all tested plants before the cold treatment (Figure 7K–P). In addition, the endogenous tangeretin levels and CHS activity were significantly reduced in the VIGS lines compared with the TRV:00 control before and after exposure to the cold conditions (Figure 7Q,R). Collectively, these findings indicate that *CiNFYA1* serves as a positive regulator of cold tolerance by modulating *CiCHS2* expression.

### 2.6 CiNFYA1 Transcriptionally Regulated and Interacted With CiGBF3

NF‐YAs have been known to exert their function by regulating or interacting with other TFs (Myers and Holt 2018; Lu et al. 2021; Tan et al. 2022). Therefore, we are curious to know whether NFYA1 could work synergistically with CiGBF3 to co‐activate its target gene, such as *CiCHS2* in this study. To this end, we first performed a bioinformatics assessment of the promoter sequence and found that the *CiGBF3* promoter contained two canonical CCAAT sequences recognised by NF‐YA1, whereas no *cis*‐acting elements recognised by GBF3 were present within the promoter of CiNFYA1. Consequently, we asked whether there is a transcriptional regulatory relationship between the two TFs. A Y1H assay was performed using CiNFYA1 prey and a bait harbouring the *CiGBF3* promoter containing the CCAAT motifs. As a result, the yeast cells transformed with the prey and the bait grew well on the selection medium supplemented with AbA, indicating that CiNFYA1 could interact with the promoter of *CiGBF3* (Figure 8A). A ChIP‐qPCR assay showed that CiNFYA1 was significantly enriched in the promoter regions containing the CCAAT elements, while no enrichment was observed in the region without a CCAAT motif (Figure 8B). Furthermore, we performed a transient activation assay by employing CiNFYA1 as an effector and a reporter constructed from the *CiGBF3* promoter. Quantitative measurements and fluorescence imaging showed that the LUC activity was significantly increased when CiNFYA1 was co‐expressed with the *CiGBF3* promoter in comparison with the control (Figure 8C–E). These results indicate that CiNFYA1 acts as a transcriptional activator of *CiGBF3*. Of note, the *CiGBF3* transcript levels in the TRV‐*CiNFYA1* plants were substantially reduced relative to the TRV:00 control, while the transcript levels of *CiNFYA1* showed no significant difference in TRV‐*CiGBF3* plants when compared to the TRV:00 control (Figure S18), further supporting the regulation of *CiGBF3* by CiNFYA1.

Next, we investigated whether CiNFYA1 could interact with CiGBF3 as well. To this end, we conducted a bimolecular fluorescence complementation (BiFC) assay and found that CiNFYA1 interacted with CiGBF3 in the nucleus (Figure 8F). Furthermore, LUC complementation imaging (LCI) assays also verified the interaction between CiNFYA1 and CiGBF3 in vivo (Figure 8G). Next, we investigated whether and how the interaction affected the activation of *CiCHS2*. CiNFYA1 and CiGBF3 were expressed, alone or together, with the LUC reporter driven by the *CiCHS2* promoter in *N. benthamiana* leaves. Interestingly, the coexpression of CiNFYA1 and CiGBF3 significantly enhanced the LUC activity in comparison with the expression of each effector alone (Figure 8H,I), implying that the interaction between CiGBF3 and CiNFYA1 led to greater activation of *CiCHS2*.

## 3 Discussion

Cold stress, a predominant environmental stressor, compromises key agronomic traits in chill‐sensitive cultivars, imposing substantial constraints on global crop productivity. Deciphering cold adaptation molecular mechanisms guides precision genome editing strategies for cold‐tolerant cultivars generation, ultimately safeguarding agricultural yield stability under temperature fluctuations (Zhang, Zhu, Gong, and Zhu 2022; Dahro et al. 2023; Yang et al. 2023). To withstand persistent environmental challenges, plants have evolved multilayered adaptive strategies, with stress‐inducible biosynthesis of various protective secondary metabolites representing a biochemical defence module that reinforces cellular homeostasis (Kaplan and Guy 2004; Zhu 2016; Xiao, Qu, et al. 2024; Xiao, Zhang, et al. 2024). Tangeretin, a polymethoxylated flavonoid, has been demonstrated to have potent free radical scavenging capacity, emerging as a potential cytoprotective modulator in plant abiotic stress adaptation (Liang et al. 2021). However, the role of tangeretin in response to cold stress remains inadequately understood, and the molecular mechanisms underlying tangeretin accumulation adapting to cold climates are still not well elucidated. In this study, we identified the function of *CiCHS2* from *C. ichangensis* in tangeretin biosynthesis and confirmed CiNFYA1 and CiGBF3 serve as upstream transcriptional activators that govern cold tolerance in citrus. Additionally, these two regulatory proteins could interact physically in a way that can work in synergy to promote *CiCHS2* expression and tangeretin synthesis. Intriguingly, CiNFYA1 acts as a transcriptional activator of *CiGBF3*, further reinforcing the transcriptional activation of *CiCHS2* in response to cold climates. This study reveals a previously unrecognised regulatory module involved in tangeretin biosynthesis that regulates cold resilience in citrus.

Accumulating evidence demonstrates that plants synthesise specialised metabolites to safeguard subcellular integrity through coordinated mechanisms, such as membrane stabilisation, ROS scavenging (Ding et al. 2019; Liu, Song, et al. 2022; Liu, Gao, et al. 2023; Wang et al. 2023). Flavonoids, a group of natural compounds with potent antioxidant, ROS scavenging and metal ion chelating properties (Peng et al. 2024), have been shown to exert positive effects on multiple environmental stresses in plants, including fungal pathogens and abiotic stresses (Mei et al. 2023; An et al. 2024; Du et al. 2024; Yang et al. 2024; Li et al. 2025; Ni et al. 2025). Tangeretin, an O‐polymethoxylated flavone of flavonoids, has been the focus of recent research in the field of human disease (Raza et al. 2020; Lv et al. 2021). Although tangeretin has been shown to be a strong antioxidant in suppressing the pathogenicity of the rice blast fungus (Liang et al. 2021), its function in the abiotic stress response of plants remains underexplored, including cold. In our previous study (Xiao, Qu, et al. 2024), we demonstrated that tangeretin accumulation is strongly induced by cold stress, with significantly higher levels of tangeretin detected in the cold‐tolerant Ichang papeda compared to the cold‐sensitive HB pummelo. Meanwhile, another research (Peng et al. 2024) also demonstrated that tangeretin is highly accumulated in wild mandarins, while remaining undetectable in modern cultivated mandarins, further supporting its potential role in cold adaptation and highlighting the differences in cold tolerance among citrus varieties. Herein, exogenous tangeretin application significantly enhanced cold tolerance via ROS scavenging, demonstrating the metabolic engineering potential for citrus cold acclimation. However, the potential for tangeretin influencing other metabolites could not be wholly excluded, as it has been shown to regulate redox homeostasis in animals by affecting signalling pathways like AMPK and mTOR (Yang et al. 2021; Wang, Zhang, et al. 2024). While these signalling pathways similarly play crucial roles in plants stress adaption (Wang, Zhao, et al. 2018; Muralidhara et al. 2021; Liu and Xiong 2022; Huang, Wang, et al. 2025), the crosstalk between tangeretin and phytokinases remains unexplored, highlighting a critical gap in plant stress signalling networks. Additionally, chalcone synthase (CHS), the rate‐limiting enzyme for flavonoid synthesis, also plays a vital role in modulating tangeretin accumulation in response to cold stress by its enzyme gene *CiCHS2* in Ichang papeda. The transcription levels of *CiCHS2* were found to be induced under cold stress, while the VIGS‐mediated gene suppression of *CiCHS2* led to a significant reduction in tangeretin synthesis and, consequently, a substantial compromise in the cold tolerance of Ichang papeda. Notably, phenotypic restoration was achieved through exogenous tangeretin application in TRV‐*CiCHS2* plants, validating the metabolite's functional necessity in citrus cryoprotective mechanisms. Although *CHS2* has been characterised as a key mediator of cold‐inducible tangeretin biosynthesis in this study, substantial evidence demonstrates that COMTs, the caffeic acid *S*‐adenosyl‐L‐methionine (SAM)‐dependent *O*‐methyltransferases, are also involved in tangeretin biosynthesis (Liao et al. 2023; Peng et al. 2024). However, their cold‐responsive regulation of tangeretin‐mediated cold response in plants remains elusive. Unlike *COMTs* (OMT3/4/5/6; homologues of *Cg5g29380*) that were strongly correlated with tangeretin accumulation during fruit development, the cold induction was attenuated, implying the presence of functional diversification of *COMTs* between development and stress adaptation (Figure S19). Interestingly, a great correlation between the induction of *CrOMT1* transcript (the *Citrus reticulata* homologue of *Cg1g005540*) and tangeretin accumulation was observed in response to UV‐B irradiation (Liu et al. 2020). Here, *Cg1g005540* was found to be strongly induced by cold stress, suggesting that it may play a potential role in the plant cold stress response. This observation warrants further investigation in the future to provide innovative and comprehensive insights into the regulatory network of tangeretin synthesis in cold stress adaptation.

Metabolic changes in plants represent the ultimate response to environmental stress, are controlled by a highly fine‐grained transcriptional regulatory network, in which transcription factor regulatory proteins serve as important bridges for translating external signals into transcriptional regulation of metabolite‐corresponding target genes (Balmer et al. 2013; Ding et al. 2020). A significant proportion of studies have demonstrated that flavonoid synthesis is predominantly governed by MYB‐bHLH‐WD40 (MBW) proteins in isolation or in a synergistic manner, and this process has been found to play a pivotal role in the regulation of floral pigmentation, plant growth and development, as well as in responses to adversity, including the response to cold stress in plants (Lepiniec et al. 2006; Agati and Tattini 2010; Xu et al. 2015; Genzel et al. 2021; Yu et al. 2022). Furthermore, other transcription factors have also been shown to regulate flavonoid synthesis through MBW‐dependent or ‐independent pathways. For instance, the bZIP protein LcABF2/3 in lychee promotes *LcMYB1*‐mediated anthocyanin accumulation during lychee fruit ripening (Hu et al. 2019), and SlHY5 in tomato directly binds to promoters such as *PAL*, *CHS1* and *CHS2* to regulate the accumulation of flavonoids under blue light (Qiu et al. 2019). These findings imply that the regulatory network of flavonoid synthesis is still underdeveloped, and in particular, the biosynthesis of specific flavonoid classes in response to adversity stress needs to be further explored. In this study, we conducted Y1H screening and successfully fished out CiGBF3 and CiNFYA1 as two candidate proteins, which were then confirmed as transcriptional activators of *CiCHS2* by conducting various analyses, including the protein–DNA interaction and dual LUC assays, and genetic transformation. To date, GBF3 protein has been less studied in abiotic stresses in plants, in which it has been reported to be widely involved in drought, cold and salt stress responses in *Arabidopsis thaliana* (Dixit et al. 2019), suggesting that GBF3 also plays an important role in plant adversity response. However, the mechanism of GBF3‐mediated stress response has not been fully elucidated. A more recent study reported that GBF3 in pear was able to modulate plant salt tolerance by regulating starch metabolism and sorbitol synthesis, further validating the role of GBF3 in abiotic stress in fruit trees (Dong et al. 2024). The current finding adds a new target gene for the GBF3 regulatory reservoir, which may shed better light on understanding the regulatory network of GBFs. The present study demonstrated that *CiGBF3* mRNA abundance was clearly induced by cold, which is consistent with the expression pattern of *CiCHS2* under cold stress, implying the predominant role in modulation of tangeretin‐mediated cold responses in Ichang papeda. This assumption was clarified by the functional validation through *CiGBF3* gain‐ and loss‐of‐function analyses, with overexpression enhancing tolerance whereas silencing significantly compromised cold tolerance. Concomitantly, *CiGBF3* overexpression elevated tangeretin accumulation, whereas VIGS‐mediated silencing depleted this synthesis. Notably, exogenous tangeretin application rescued cold sensitivity in *CiGBF3*‐silenced plants, demonstrating the essential role of tangeretin in *CiGBF3*‐mediated cryoprotection. These results provide further compelling evidence that sheds light on the function of tangeretin in cold responses, while indicating the crucial role of CiGBF3 in modulation of *CiCHS2* transcription activity and tangeretin accumulation under cold stress.

NF‐Y transcription factors, through interactions of the NF‐YA/B/C complex, the NF‐YB/C/X heterotrimer, or individual NF‐Y subunits with various transcription factors, have been demonstrated to play crucial roles in a wide array of biological processes in plants, including the regulation of plant growth and development as well as the response to stressors. Recent studies have demonstrated that NF‐Ys are well‐documented mediators of drought adaptation through both ABA‐dependent and ‐independent pathways (Wang, Wei, et al. 2022; Wang, Mao, et al. 2024). However, their mechanistic contributions to plant cold stress adaptation remain underexplored. In this study, NFYA1 is identified as an upstream positive regulator of *CiCHS2*‐mediated cold tolerance in *C. ichangensis*, specifically binding to the CCAAT cis‐element in the *CiCHS2* promoter. VIGS‐induced knockdown of *CiNFYA1* significantly decreased the expression of *CiCHS2*, leading to compromised cold tolerance in Ichang papeda by disrupting *CiCHS2*‐mediated ROS homeostasis. Furthermore, NF‐Y and bZIP proteins have been shown to form heterodimers to regulate the expression of target genes (Myers and Holt 3rd 2018). Not surprisingly, our findings demonstrate that CiNFYA1 and CiGBF3 form a functional heterodimer in *C. ichangensis*, synergistically enhancing *CiCHS2* promoter activation through direct transcriptional cooperativity. More interestingly, CiNFYA1 functions as an upstream transcriptional activator of *CiGBF3* by directly binding to the CCAAT cis‐element in its promoter, implying the cascade role of CiNFYA1 within the CiNFYA1‐CiGBF3‐*CiCHS2* module that confers cold resilience in *C. ichangensis*. Accumulating evidence suggests that ABA plays a pivotal role in mediating adaptation to cold stress by coordinating stress signalling and regulating the transcription of some *Cold‐Responsive* (*COR*) genes (Waadt 2020; Ding et al. 2024). In tomato, cold‐induced ABA production activated the expression of *AREB4*, leading to the upregulation of *TPS9* and promotion of trehalose biosynthesis to enhance cold tolerance (Liu, Wang, et al. 2023). Furthermore, ABA was found to upregulate the expression of *bZIP73* ^*jap*^, which exhibited transcriptional activation of peroxidase genes, leading to cold tolerance in rice (Liu et al. 2018). We also observed that the transcript levels of *CiNFYA1*, *CiGBF3* and *CiCHS2* were significantly induced by ABA, implying the notion that the CiNFYA1/CiGBF3‐*CiCHS2* module may be governed by ABA in response to cold stimuli (Figure S20). Further study will be valuable in investigating whether ABA signalling cooperates with the CiNFYA1/CiGBF3‐*CiCHS2* module to regulate tangeretin‐mediated cold tolerance, thereby offering new insights into the roles of hormone signalling and flavonoids in enhancing cold stress tolerance.

**FIGURE 9:**
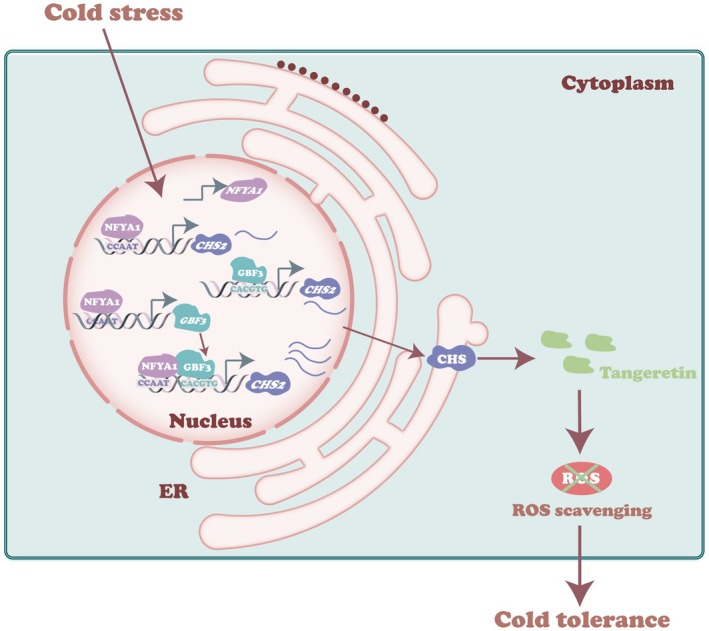
Working model illustrating the role of CiNFYA1–CiGBF3–*CiCHS2* in cold tolerance. Cold stress leads to upregulation of both CiNFYA1 and CiGBF3, which can in turn directly regulate and activate *CiCHS2* through binding to the CCAAT and G‐box elements, respectively, within the promoter region. In addition, CiNFYA1 transcriptionally activates *CiGBF3*, leading to a hierarchical regulatory cascade on *CiCHS2*. Meanwhile, CiGBF3 interacts with CiNFYA1 to form a heterodimeric complex, leading to more robust upregulation of *CiCHS2*. As a result, *CiCHS2* is prominently induced to elevate the biosynthesis of tangeretin, which plays a role in conferring enhanced cold tolerance. ER, endoplasmic reticulum.

Based on our findings and previous results, we propose a hierarchical regulatory module comprising CiNFYA1/CiGBF3‐*CiCHS2* governing cold‐inducible tangeretin biosynthesis (Figure 9). Cold stress results in upregulation of *CiNFYA*1 and *CiGBF3* at transcriptional levels. As a result, they independently regulate *CiCHS2* by interacting with the corresponding cis‐acting element within the promoter, triggering *CiCHS2‐*mediated tangeretin synthesis. Meanwhile, the cold‐induced CiNFYA1 also regulates *CiGBF3* to amplify the *CiGBF3*‐*CiCHS2* transcriptional regulation. In addition, CiNFYA1 and CiGBF3 form a protein complex, which in turn further activates *CiCHS2*, leading to enhanced cold tolerance via the tangeretin‐mediated ROS scavenging. Taken together, our findings unravel the regulatory mechanism governing the cold‐inducible tangeretin biosynthesis, which provides new insights into the potential targets that can be molecularly engineered to enhance cold tolerance in plants.

## 4 Materials and Methods

### 4.1 Plant Materials and Growth Conditions

Seeds of wild‐type Ichang papeda (*Citrus ichangensis*), HB pummelo ( *C. grandis* cv. Hirado Buntan) and lemon ( *C. limon* ) obtained from the Citrus Breeding Center at Huazhong Agricultural University (Wuhan, China), were cultivated in standard potting substrate under controlled phytotron conditions (25°C, 16/8 h light/dark photoperiod) to germinate. For analyses of gene expression, the 3‐month‐old seedlings of Ichang papeda and HB pummelo were then transferred to a growth chamber set at 4°C, and leaves were collected at 0, 6, 12, 24, 72 and 120 h, frozen immediately in liquid nitrogen and kept at −80°C until further use. In order to analyse the effect of exogenous tangeretin on cold response, 2‐month‐old HB pummelo and lemon seedlings were treated or not with 500 μM tangeretin for 2 days before exposure at −4°C for 6 h. Methanol (MeOH, 10%, v/v) served as the solvent control, followed by growth recovery for 1 day at 25°C.

### 4.2 RNA Extraction and Quantitative Real‐Time RT‐PCR Analysis

Total RNA was extracted from samples using an RNA extraction kit (RN33; Aidlab Biotech Co. Ltd., Beijing, China), followed by complementary DNA (cDNA) synthesis with HiScript III Reverse Transcriptase (R323, Vazyme, Nanjing, China). RT‐qPCR was performed using the ChamQ Universal SYBR qPCR Master Mix (Q711, Vazyme, Nanjing, China) according to the manufacturer's instructions, with *Actin* as the endogenous control. Gene expression levels were normalised using the 2^−ΔΔCT^ method (Livak and Schmittgen 2001). All samples were analysed in biological triplicates. Primer sequences are detailed in Table S2.

### 4.3 Histochemical Assay of GUS Activity

Histochemical assay of β‐glucuronidase (GUS) activity was carried out using transient expression in sweet orange ( *Citrus sinensis* ) embryogenic callus. The 1125‐bp *CiCHS2* promoter sequence (p*CHS2*), spanning the genomic region upstream of the translation start site from *C. ichangensis*, was PCR‐amplified using genome‐specific primers and directionally cloned into the DX2181G vector containing a GUS gene, generating the p*CHS2*:GUS fusion construct. Callus was transformed with either p*CHS2*: GUS construct or the DX2181G empty vector (Zhang, Ming, Khan, et al. 2022) and cocultured for 72 h at 25°C under dark conditions, followed by culturing for 3 days at 4°C. Histochemical staining of the callus was performed by the GUS staining kit (Cat. SL7160; Coolaber, Beijing, China) following the manufacturer's protocol.

### 4.4 Gene Isolation and Analysis

The published RNA‐seq data in an earlier study (Xiao, Qu, et al. 2024) was analysed to confirm the genes in the tangeretin biosynthesis pathway upregulated by cold stress. Full‐length coding sequences of *CiCHS2* (1176 bp), *CiGBF3* (1266 bp) and *CiNFYA1* (1098 bp) were amplified from *Citrus ichangensis* genomic DNA by PCR following the manufacturer's protocol (Cat. P505; Vazyme, Nanjing, China), with all amplicons sequence‐verified by sequencing. Protein sequence homology was assessed using DNAMAN for multiple sequence alignment. Phylogenetic reconstruction of CiGBF3 and CiNFYA1 orthologs was conducted using MEGA X.

### 4.5 Subcellular Localisation Analysis

Full‐length coding sequences (CDS) of *CiCHS2*, *CiGBF3* and *CiNFYA1* (termination codon excluded) were directionally cloned into the p101LYFP expression vector driven by the CaMV 35S promoter, generating fusion constructs. *Agrobacterium* strain *GV3101* was transformed with either recombinant plasmids or the empty vector control. Transformed colonies were cultured overnight in LB medium supplemented with 200 μM acetosyringone, pelleted by centrifugation and resuspended in infiltration buffer (10 mM MgCl_2_ and 10 mM MES, pH 5.7) to an optical density (OD600) of 1.0. The CiCHS2‐YFP and CiNFYA1/CiGBF3‐YFP constructs were coexpressed alongside organelle‐specific markers (35S: OFP‐HDEL for the endoplasmic reticulum (ER) and 35S: mCherry for nuclear localisation), along with p19, in a 1:1:1 volume ratio. Bacterial suspensions were transiently transfected into the leaves of *Nicotiana benthamiana*. Confocal laser scanning microscope (Leica TCS‐SP8, Leica Microsystems, Wetzlar, Germany) was performed for the fluorescence signals observation with a 514‐nm laser line and detected from 530 to 560 nm at 2–3 days post‐infiltration.

### 4.6 Transcriptional Activation Activity Analysis

Transcriptional activation activity assays were conducted using the firefly luciferase (LUC) reporter systems. In brief, the *CiNFYA1/CiGBF3* CDS was cloned into the pBD vector to generate effector constructs, while the reporter comprised pGreenII 0800‐LUC with 5 × GAL4 upstream of the 35S::LUC cassette. The pBD empty vector was utilised as the negative control, whereas the pBD‐VP16, the pBD vector containing the LexA DNA‐binding domain‐VP16 activation domain, was employed as a positive control. *Agrobacterium tumefaciens* GV3101 transformed with these constructs via heat shock was infiltrated into *N. benthamiana* leaves. After 72 h of dark incubation at 25°C, leaves were sprayed with 0.1 mM D‐luciferin potassium salt (Coolaber CL6930, China) and imaged using NightSHADE LB 985 (Berthold, Germany) with Indigo software. Quantitative LUC activity was measured with the Dual Luciferase Reporter Assay Kit (Cat. E1910; Promega, Madison, WI, USA) on an Infinite 200 Pro microplate reader (Tecan, Mannedorf, Switzerland).

### 4.7 Vector Construction and Transformation

To generate transgenic plants, full‐length CDS of *CiCHS2* and *CiGBF3* were directionally cloned into the binary vector pGWB411 via the gateway recombination technology (Invitrogen, Carlsbad, USA), placing transgenes under the control of the CaMV35S promoter. Recombinant plasmids were transformed into *A. tumefaciens* strain *GV3101* for subsequent tobacco (*Nicotiana nudicaulis*) transformation following previous reports (Huang et al. 2013; Ming et al. 2021). Transgenic plants were selected on the MS medium (Coolaber, Beijing, China) containing 50 μg/mL kanamycin. Genomic integration was confirmed through endpoint PCR using transgene‐specific primers, while transcriptional activation was verified by qRT‐PCR. Transgenic tobacco plants at the T2 generation were advanced for further analysis.

The CRISPR‐Cas9 genome editing vector was constructed as previously described and subsequently introduced into the *Agrobacterium tumefaciens* strain EHA105 (Zhang et al. 2021). In brief, the different sgRNAs (with the primers for sgRNA synthesis listed in Table S2) were ligated to an AtU6‐26‐sgRNA‐SK vector and then transferred to the proYAO‐Cas9‐NOS binary vector. The plant transformation of Ichang papeda was according to a previous report (Huang et al. 2013).

### 4.8 Virus‐Induced Gene Silencing (VIGS)

Targeted gene silencing in *C. ichangensis* was achieved using a tobacco rattle virus (TRV)‐mediated VIGS system, as previously optimised (Xiao, Qu, et al. 2024). In brief, gene‐specific fragments of the targets (*CiCHS2*, containing 54 bp 5′‐UTR and 246 bp CDS; *CiGBF3*, 306 bp CDS; *CiNFYA1*, 306 bp CDS) were inserted into the TRV2 vector, followed by transformation into *Agrobacterium tumefaciens* strain *GV3101*. Mixed suspensions of TRV1 and recombinant TRV2 (1:1 v/v, OD600 = 1.0) were infiltrated into the 30‐day‐old *C. ichangensis* albino shoots following established protocols (Dai et al. 2018; Wang et al. 2019). Systemic leaves were harvested 30 days post‐infiltration for molecular validation: genomic PCR confirmed TRV vector integration, while RT‐qPCR quantified transcript reduction degree. The seedlings with target gene suppression were advanced for phenotypic analysis. Primer sequences are catalogued in Table S2.

### 4.9 Library Screening and Yeast‐One‐Hybrid (Y1H) Assay

A 1125‐bp *CiCHS2* promoter fragment was PCR‐amplified and directionally cloned into the pAbAi bait vector using *BamHI/XhoI* restriction sites. The recombinant construct was transformed into the Y1HGold strain for bait–reporter integration. cDNA library screening was performed using the Matchmaker Gold Yeast‐One‐Hybrid System (Clontech 630491, USA) under stringent selection with 50 ng/mL aureobasidin A (AbA). Putative transcription factor (TF)‐DNA interactors were isolated through colony PCR amplification (Rapid Taq Master Mix, P222‐03, Vazyme, China) and sequencing. Candidate TF sequences were annotated against the *Citrus* Pan‐Genome to Breeding Database (CPBD, http://citrus.hzau.edu.cn/) (Liu, Wang, et al. 2022) for functional characterisation.

To validate the binding of CiNFYA1 and CiGBF3 to the *CiCHS2* promoter, as well as the binding of CiNFYA1 to the *CiGBF3* promoter, full‐length coding sequences (CDS) of the two transcription factors were cloned into the *NdeI/EcoRI* restriction sites of the pGADT7 prey vector. A 1125‐bp *CiCHS2* promoter fragment (P1) harbouring conserved G‐box (CACGTG) and CCAAT cis‐elements and the *CiGBF3* promoter were ligated into the pAbAi bait vector. Site‐directed mutagenesis via overlap extension PCR separately generated mutant variants with mutations of G‐box (from CACGTG to AAAAAA) and CCAAT (from TCCAAT to AAAAAA). Bait–prey pairs, along with the negative control (pGADT7‐AD+baits) and positive control (pGAD‐p53+ p53‐AbAi), were combined and cotransformed into the yeast Y1HGold strain. Y1H assay was performed following the Matchmaker Gold Yeast Transformation System 2 protocol (Cat. 630439, Clontech, USA).

### 4.10 Electrophoretic Mobility Shift (EMSA) Assay

CiNFYA1 and CiGBF3 CDS were cloned into the pHMGWA expression vector to get fusion plasmids His‐CiNFYA1 and His‐CiGBF3, which were expressed in the *Escherichia coli* Rosetta (DE3) competent cells (Weidi Biotechnology, China). The fused proteins were induced by 0.5 mM isopropyl‐β‐D‐1‐thiogalactopyranoside (IPTG), followed by affinity purification using Ni‐NTA agarose (Qiagen 30 230, Germany). Biotinylated DNA probes containing original *CiCHS2* promoter motifs (G‐box: 5′‐CACGTG‐3′; CCAAT: 5′‐TCCAAT‐3′) or mutated variants (5′‐AAAAAA‐3′) were synthesised. Unlabelled fragments were used as the DNA competitor. Protein‐DNA interactions were resolved using the Chemiluminescent EMSA Kit (Beyotime GS009, China), and the signal was visualised by chemiluminescence imaging (Tanon 5200, China). Probe designs are detailed in Table S2.

### 4.11 ChIP‐qPCR Assay

Transient expression of *CiNFYA1* and *CiGBF3* in sweet orange callus was carried out as previously described (Ming et al. 2021). The full‐length *CiNFYA1* and *CiGBF3* coding sequences were cloned into the pGWB405 binary vector to generate GFP‐tagged fusions. The 35S:CiNFYA1‐GFP and 35S:CiGBF3‐GFP constructs were transformed into *Agrobacterium* strain *GV3101* for callus transformation. Transiently transformed calli were harvested 72 h post‐coculture (25°C, darkness) for ChIP‐qPCR analysis. Firstly, callus tissue (2 g) from 35S:CiNFYA1‐GFP/35S:CiGBF3‐GFP‐overexpressing plants was cross‐linked with 1% (w/v) formaldehyde and quenched with 125 mM glycine. Chromatin was fragmented to 200 to 1000‐bp by sonication (Bioruptor plus, Belgium) and immunoprecipitated using anti‐GFP magnetic beads (MBL D153‐10, Japan), with wild‐type chromatin as the negative control. The purified DNA was dissolved in distilled water and used for qPCR with specific primers. The fold enrichment of DNA fragments was calculated according to Bowler et al. (2004). Data are presented as means ± SD of three biological replicates.

### 4.12 Dual LUC Assay

Dual LUC assays using *N. benthamiana* leaves were performed as described previously (Hellens et al. 2005). Effector constructs encoding full‐length *CiNFYA1* and *CiGBF3* CDS were cloned into the pGREEN II‐62‐SK vector, while reporter constructs included original *CiCHS2* promoter fragments (containing G‐box [CACGTG] and CCAAT [TCCAAT] motifs) or their mutated variants (AAAAAA), respectively, generated by overlap extension PCR, alongside a *CiGBF3* promoter, all ligated into pGREEN II‐0800‐LUC. Effector–reporter mixtures were agroinfiltrated into leaves of *N. benthamiana* plants, cultured for 2–3 days under long‐day conditions (16 h light/8 h dark, 25°C). For the measurement of LUC activity, leaf samples were analysed using the Dual Luciferase Reporter Assay Kit (Cat. E1910; Promega, Madison, WI, USA) in accordance with the manufacturer's protocol. Fluorescence signal intensity was visualised by infiltrating the leaves with 0.1 mM D‐luciferin potassium salt (Coolaber CL6930, China) followed by luminescence detection using NightSHADE LB 985 (Berthold, Germany) with Indigo software. Three biological replicates were measured for each sample.

### 4.13 Bimolecular Fluorescence Complementation (BiFC) Assay

Full‐length CDS of CiNFYA1 and CiGBF3 were separately cloned into the N‐terminal and C‐terminal of YFP‐101, generating nYFP‐CiGBF3 and CiNFYA1‐cYFP expression vectors. *Arabidopsis thaliana* AtbZIP63 fused to nYFP and cYFP served as an interaction‐positive control (Walter et al. 2004). Recombinant constructs were transformed into *A. tumefaciens* strain *GV3101* and coinfiltrated (1:1 volumetric ratio) *N. benthamiana* young leaves. YFP fluorescence signals were monitored 48 h post‐infiltration using a Leica TCS‐SP8 confocal microscope (Leica TCS‐SP8; Leica Microsystems, Wetzlar, Germany) with 514 nm excitation and sequential emission capture (530–560 nm, HyD detectors).

### 4.14 LUC Complementation Imaging

Full‐length *CiGBF3* and *CiNFYA1* coding sequences were directionally cloned into the JW771 (nLUC) and JW772 (cLUC) vectors, respectively, for N‐ and C‐terminal luciferase fusions. The recombinant plasmids were introduced into *A. tumefaciens* strain *GV3101* and co‐infiltrated into *Nicotiana benthamiana* leaves, followed by cultured under long‐day photoperiod (16‐h light/8‐h dark) at 25°C for 48 h prior to substrate spray with 0.1 mM D‐luciferin potassium salt (Coolaber CL6930, China). Luminescence signals were detected using a NightSHADE LB 985 in vivo imaging system (Berthold Technologies, Germany) with Indigo software.

### 4.15 Cold Tolerance Assays

Four‐week‐old/7‐week‐old transgenic tobacco plants overexpressing *CiCHS2/CiGBF3*, along with wild‐type (WT) controls, were maintained in a growth chamber at 2°C for 2 h, subsequently exposed to −2°C until the onset of cold damage phenotypes emerged, and then allowed to recover for 24 h at 25°C. TRV2‐*CiCHS2*/TRV2‐*CiNFYA1* and TRV control plants, pretreated with Methanol (MeOH, 10%, v/v) or 500 μM tangeretin for 2 days, were exposed at −4°C for a period of time when freezing damage was observed, followed by recovery for 24 h at 25°C. In another experiment, *CiNFYA1*‐VIGS and TRV control seedlings were subjected to −4°C until the onset of cold damage phenotypes emerged, and then recovered for 24 h at 25°C. Additionally, CRISPR‐Cas9‐edited lines were assessed for cold tolerance using the identical stress protocol. Phenotypes and chlorophyll fluorescence imaging were photographed before and after the experiment, and leaf samples were collected at the beginning and conclusion of the cold treatment for subsequent analysis.

### 4.16 Physiological Measurement and Histochemical Staining

Chlorophyll fluorescence imaging was captured using an IMAGINGPAM chlorophyll fluorimeter (Walz, Germany), while *Fv*/*Fm* ratios were acquired via ImagingWin software. Electrolyte leakage (EL) was measured as described previously (Ming et al. 2021). Total protein and MDA content were measured by using the corresponding detection kits (Cat. A405 for protein; Cat. A003 for MDA, Nanjing Jiancheng Bioengineering Institute, China) as instructed by the manufacturer. CHS activity and tangeretin content were measured by using specific detection kits based on competitive enzyme‐linked immunosorbent assay (ELISA) method (Cat. A‐P0212A for enzyme activity, Jiangsu Meibiao Biotechnology Co. Ltd., China; Cat. YJ980023 for tangeretin, Shanghai Enzyme‐linked Biotechnology Co. Ltd., China), following the manufacturer's guidelines. To determine the accumulation of H_2_O_2_ and O_2_˙^−^, histochemical staining was conducted using 3,3′‐diaminobenzidine (DAB) and nitrotetrazolium blue chloride (NBT), respectively, following the methods outlined by Huang et al. (2013).

### 4.17 Statistical Analysis

All data represent three biological replicates analysed via one‐way analysis of variance (ANOVA) with Student's *t*‐tests (SPSS 26.0, IBM, NY, USA). Results are expressed as mean ± SD (standard deviation) with significance thresholds of *p < 0.05, **p < 0.01 and ***p < 0.001.

### 4.18 Accession Numbers

Sequence data from this article are available in the reference genome of *C. ichangensis* in the CPBD (Citrus Pan‐genome to Breeding Database, http://citrus.hzau.edu.cn/): *CiCHS2*, Ci190150; *CiGBF3*, Ci000230; *CiNFYA1*, Ci076120.

## Author Contributions

J.‐H.L. and P.X. conceived and designed the research. P.X. performed the experiments and analysed the data, with the assistance of J.Q., Y.Z., W.X., T.F. and X.Z. Y.W. assisted with bioinformatics analysis. P.X. wrote the manuscript draft. C.L. and J.‐H.L. finalised the writing and revision of the manuscript.

## Conflicts of Interest

The authors declare no conflicts of interest.

## Supporting information


**Table S1:** Potential *CiCHS2* regulating proteins identified by Y1H screening.
**Table S2:** List of primers used in this study.

**Figure S1:** The relative tangeretin content of HB pummelo (HB) and Ichang papeda (Yi Chang Cheng, YCC) under cold treatment.
**Figure S2:** Influence of different tangeretin concentrations on cold tolerance of HB pummelo.
**Figure S3:** Exogenous tangeretin application enhanced cold tolerance of lemon.
**Figure S4:** Molecular identification of the *CiCHS2*‐VIGS plants.
**Figure S5:** Molecular identification of the *CiCHS2*‐overexpression plants.
**Figure S6:** Overexpression of *CiCHS2* enhanced cold tolerance of transgenic tobacco.
**Figure S7:** Partial PCR identification results of Y1H screening.
**Figure S8:** Expression patterns of *CiNFYA1* and *CiGBF3* under cold treatment.
**Figure S9:** Phylogenetic analysis of CiNFYA1 and all NF‐Y transcription factors from *Arabidopsis*.
**Figure S10:** Phylogenetic analysis of *CiGBF3* and all bZIP transcription factors from *Arabidopsis*.
**Figure S11:** Subcellular localization and transcriptional activation activity of CiGBF3 and CiNFYA1.
**Figure S12:** Molecular identification of the *CiGBF3*‐overexpression plants.
**Figure S13:** The expression levels of *CiCHS2* in overexpressing‐*CiGBF3* plants.
**Figure S14:** Molecular identification of the TRV2‐*CiGBF3* plants.
**Figure S15:** The expression levels of *CiCHS2* in TRV‐*CiGBF3* plants.
**Figure S16:** Molecular identification of the TRV2‐*CiNFYA1* plants.
**Figure S17:** The expression levels of *CiCHS2* in TRV‐*CiNFYA1* plants.
**Figure S18:** The expression levels of *CiGBF3 and CiNFYA1* in TRV:00 and VIGS plants.
**Figure S19:** Expression analysis of *COMTs* in Ichang papeda under cold treatment.
**Figure S20:** The expression of *CiNFYA1*, *CiGBF3 and CiCHS2* in response to ABA.

## Data Availability

The data supporting the findings of this study are available in the article and its Supporting Information files.
