# Supplementary material for: The Transcription Factors NFYA1 and GBF3 Jointly Regulate CHS2 to Promote Tangeretin Accumulation and Cold Tolerance in Citrus
Source: Plant Biotechnol J. 2025 Sep 16;24(2):582–601. doi: 10.1111/pbi.70371 (PMC12906813; doi:10.1111/pbi.70371)
Supplement: Supplementary file 1 — Table S1: Potential CiCHS2 regulating proteins identified by Y1H screening. Table S2: List of primers used in this study. [file PBI-24-582-s001.docx]

Supplemental Tables:

**Supplemental Table S1.** Potential *CiCHS2* regulating proteins identified by Y1H screening.

| Gene ID | Discription |
| --- | --- |
| Ci076120 | nuclear transcription factor Y subunit A-1 |
| Ci000230 | G-BOX-BINDING FACTOR 3 |
| Ci048440 | ethylene-responsive transcription factor ERF073 |
| Ci263940 | NAC domain-containing protein 2-like (NAC) |
| CI002910 | transcription factor UNE10, PIF8 |
| Ci108610 | zinc finger A20 and AN1 domain-containing stress-associated protein 8 |
| Ci077360 | probable calcium-binding protein CML45 |
| Ci243550 | EARLY RESPONSIVE TO DEHYDRATION 15 |
| Ci136820 | ADP-ribosylation factor |
| Ci226570 | protein BRASSINAZOLE-RESISTANT 1 |
| Ci095680 | coiled-coil domain-containing protein 9 |
| Ci262830 | zinc finger protein CONSTANS-LIKE 4, BBX5 |
| Ci288460 | transcription initiation factor TFIID subunit 10 |
| Ci164020 | homeobox protein HD1, KNOX2 |
| Ci209920 | COR15a |
| Ci044120 | aldehyde dehydrogenase family 2 member B7 |
| Ci000370 | SUPPRESSOR OF NPR1-1 |
| Ci178820 | auxin-binding protein ABP19a |
| Ci010950 | LIM domain-containing protein WLIM2B |

**Supplemental Table S2.** List of primers used in this study.

| **Primer name** | **Primers sequence (5'-3')** |
| --- | --- |
| qPCR-*Actin*-F | CCGACCGTATGAGCAAGGAAA |
| qPCR-*Actin*-R | TTCCTGTGGACAATGGATGGA |
| qPCR-*CiCHS2*-F | TCGAGCGTCCGTTGTATCAG |
| qPCR-*CiCHS2*-R | TTTGAGATCAAGCCGGGGAC |
| qPCR-*CiNFYA1*-F | CTTCTCAACCTTGGTGGCGT |
| qPCR-*CiNFYA1*-R | ATTTCCTTGGTTGCATCGGC |
| qPCR-*CiGBF3*-F | GAGTGCACAACTGGGAAACAAG |
| qPCR-*CiGBF3*-R | AAGCTGATGTAGCTTGGCACC |
| pro*CiCHS2*-F | GAAGAGTAGAATTTGATTAC |
| pro*CiCHS2*-R | TTCAAGTTATACCCTTAATGAG |
| pro*CiCHS2*-DX2181-F | CTACAGCGCTAAGCTTGGCTGCAGGAAGAGTAGAATTTGATTAC |
| pro*CiCHS2*-DX2181-R | AAGGGACTGACCACCCGGGATCCTTCAAGTTATACCCTTAATGAG |
| *CiCHS2*-F | ATGGCAACCGTTCAAGACATCAGAA |
| *CiCHS2*-R | TCAAGCTTTGATGGGGACACTGTG |
| YFP-CiCHS2-F（EcoRI） | GGATCTACTAGTGAATTCATGGCAACCGTTCAAGACAT |
| YFP-CiCHS2-R（BamHⅠ） | GGTACCGTCGACGGATCCAGCTTTGATGGGGACACTGT |
| YFP-CiGBF3-F（EcoRI） | GGATCTACTAGTGAATTCATGGGGAACAATGAAGATGGA |
| YFP-CiGBF3-R（BamHⅠ） | GGTACCGTCGACGGATCCGCCTGCAGCCACGGCA |
| YFP-CiNFYA1-F（EcoRI） | GGATCTACTAGTGAATTCATGTTACGAAGCATGC |
| YFP-CiNFYA1-R（BamHⅠ） | GGTACCGTCGACGGATCCTTTGATGGTCAGGGCCC |
| pDONR221-*CiCHS2*-F | GGGGACAAGTTTGTACAAAAAAGCAGGCTTA ATGGCAACCGTTCAAGACAT |
| pDONR221-*CiCHS2*-R | GGGGACCACTTTGTACAAGAAAGCTGGGTT AGCTTTGATGGGGACACTGT |
| pDONR221-*CiGBF3*-F | GGGGACAAGTTTGTACAAAAAAGCAGGCTTA ATGGCAACCGTTCAAGACAT |
| pDONR221-*CiGBF3*-R | GGGGACCACTTTGTACAAGAAAGCTGGGTT AGCTTTGATGGGGACACTGT |
| pDONR221-*CiNFYA1*-F | GGGGACAAGTTTGTACAAAAAAGCAGGCTTA ATGTTACGAAGCATGC |
| pDONR221-*CiNFYA1*-R | GGGGACCACTTTGTACAAGAAAGCTGGGTT TTTGATGGTCAGGGCCC |
| RNAi-*CiCHS2*-F | GGGGACAAGTTTGTACAAAAAAGCAGGCT TGCACACTCACCAAGCACGA |
| RNAi-*CiCHS2*-R | GGGGACCACTTTGTACAAGAAAGCTGGGT GTTGGGGTTTTCCTTCAAAA |
| CRISPR/Cas9-*CiCHS2*-gRNA1-F | attgGCCTGCCCACAGTGTCAACC |
| CRISPR/Cas9-CiCHS2-gRNA1-R | aaacGGTTGACACTGTGGGCAGGC |
| CRISPR/Cas9-CiCHS2-gRNA2-F | attgTGTCTGTTCGGAGATCACGG |
| CRISPR/Cas9-CiCHS2-gRNA2-R | aaacCCGTGATCTCCGAACAGACA |
| pTRV2-*CiCHS2*-F（BamHI） | AGAAGGCCTCCATGGGGATCCTGCACACTCACCAAGCACGA |
| pTRV2-*CiCHS2*-R（SmaI） | TGTCTTCGGGACATGCCCGGGGTTGGGGTTTTCCTTCAAAA |
| pTRV2-*CiGBF3*-F（BamHI） | AGAAGGCCTCCATGGGGATCCAATATTCATATGTATACT |
| pTRV2-*CiGBF3*-R（SmaI） | GTCTTCGGGACATGCCCGGGTGCATTTCCTGACGACTT |
| pTRV2-*CiNFYA1*-F（BamHI） | AGAAGGCCTCCATGGGGATCCATGTTACGAAGCATGC |
| pTRV2-*CiNFYA1*-R（SmaI） | TGTCTTCGGGACATGCCCGGGAGCATTTCCTTGGTTGC |
| pAbAi-pro*CiCHS2*-F | CTTGAATTCGAGCTCGGTACCGAAGAGTAGAATTTGATTAC |
| pAbAi-pro*CiCHS2*-R | ATACAGAGCACATGCCTCGAGTTCAAGTTATACCCTTAATGAG |
| mGBF3-pAbAi-pro*CiCHS2*-F | GCAGCCTAACACTATCTTTTTTGTCAGCATCTGCCCGT |
| mGBF3-pAbAi- pro*CiCHS2*-R | ACGGGCAGATGCTGACAAAAAAGATAGTGTTAGGCTGC |
| mNFYA1-pAbAi-pro*CiCHS2*-F | AGATCCATTTAATATGTAAAAAATACCGCATTACAAGTAC |
| mNFYA1-pAbAi- pro*CiCHS2*-R | GTACTTGTAATGCGGTATTTTTTACATATTAAATGGATCT |
| pAbAi-pro*CiGBF3*-F | CTTGAATTCGAGCTC GGTACCGTGATGTTATTGTGTTAAGT |
| pAbAi-pro*CiGBF3*-R | ATACAGAGCACATGC CTCGAGGGCCAACCACCAAACTGGAA |
| *CiGBF3*-F | ATGGGGAACAATGAAGATGGAAAGTCC |
| *CiGBF3*-R | TCAGCCTGCAGCCACG |
| *CiNFYA1*-F | ATGTTACGAAGCATGCACCA |
| *CiNFYA1*-R | TCATTTGATGGTCAGGGCC |
| SK-CiGBF3-F（BamHI） | CGCTCTAGAACTAGTGGATCCATGGGGAACAATGAAGATGGA |
| SK-CiGBF3-R（EcoRI） | GATAAGCTTGATATCGAATTCTCAGCCTGCAGCCACG |
| SK-CiNFYA1-F（BamHI） | CGCTCTAGAACTAGTGGATCCATGTTACGAAGCATGC |
| SK-CiNFYA1-R（EcoRI） | GATAAGCTTGATATCGAATTCTCATTTGATGGTCAGG |
| 0800-pro*CiCHS2*-F（HindIII） | GTCGACGGTATCGATAAGCTTGAAGAGTAGAATTTGA |
| 0800-pro*CiCHS2*-R（BamHI） | CGCTCTAGAACTAGTGGATCCTTCAAGTTATACCCTT |
| 0800-pro*CiGBF3*-F（HindIII） | GTCGACGGTATCGATAAGCTTGACGCTCTTGTGATGTTATTGTG |
| 0800-pro*CiGBF3*-R（BamHI） | CGCTCTAGAACTAGTGGATCCGGCCAACCACCAAACTGG |
| pBD-CiGBF3-F (AgeI) | AGTTGACTGTATCGCCGACCGGTATGGGGAACAATGAAGATGGA |
| pBD-CiGBF3-R (StuI) | TAATGAAACCAGAGTTAAAGGCCTTCAGCCTGCAGCCACG |
| pBD-CiNFYA1-F (AgeI) | AGTTGACTGTATCGCCGACCGGTATGTTACGAAGCATGC |
| pBD-CiNFYA1-R (StuI) | TAATGAAACCAGAGTTAAAGGCCTTCATTTGATGGTCAGG |
| *CHS2*-ChIP-CCAAT-F | CCATTTAATATGTTCCAATTACCGC |
| *CHS2*-ChIP-CCAAT-R | TATGCCGCATGAATCCTCTC |
| *CHS2*-ChIP-G-box-F | GGAGAGAGATATGTGGCGCA |
| *CHS2*-ChIP-G-box-R | TTGGTGAGCGTGCACTAACT |
| *CHS2*-CHIP-FU | TGACCATGCAATCGACCTCC |
| *CHS2*-CHIP-FU | GTTTTTGGCCATCTTAACAAAAGGA |
| ChIP-NG-F1-F | GCTGTTTGGCAATGCC |
| ChIP-NG-F1-R | GGCCAGTCATTGACGAG |
| ChIP-NG-F2-F | AGCCAGTCGTGGATTCCAAA |
| ChIP-NG-F2-R | ACCCTCATGTGGCTTACGTG |
| ChIP-NG-F1+2-F | CGTGGCTAAGGAACCCCAAT |
| ChIP-NG-F1+2-R | CCCTCATGTGGCTTACGTGT |
| ChIP-NG-EV-F | GGCCAACTCAACAACCAACC |
| ChIP-NG-EV-R | GGCTCGGCATTCTCTCGTTA |
| *CHS2*-CACGTG probe | GCAGCCTAACACTATCCACGTGGTCAGCATCTGCCCGT |
| *CHS2*-mCACGTG probe | GCAGCCTAACACTATCTTTTTTGTCAGCATCTGCCCGT |
| *CHS2*-CCAAT probe | AAAGATCCATTTAATATGTTCCAATTACCGCATTACAAGTACTAAA |
| *CHS2*-mCCAAT probe | AAAGATCCATTTAATATGTAAAAAATACCGCATTACAAGTACTAAA |
| NF-YA1-*GBF3*-probe1 | AAGCGTGGCTAAGGAACCCCAATACCCATATTGCTCGTCAA |
| mNF-YA1-*GBF3*-probe1 | AAGCGTGGCTAAGGAACAAAAAAACCCATATTGCTCGTCAA |
| NF-YA1-*GBF3*-probe2 | CTAGTTAATTGTTTAATCCAATTGAAAAAATCCGAATTAT |
| mNF-YA1-*GBF3*-probe2 | CTAGTTAATTGTTTAATAAAAAAGAAAAAATCCGAATTAT |
| L101YCE-CiNFYA1-F(EcoRⅠ) | ATGGGATCTACTAGTGAATTC ATGTTACGAAGCATGC |
| L101YCE-CiNFYA1-R(KpnⅠ) | TGGGTACATCCCGGGGGTACC TTTGATGGTCAGGGCCC |
| L101YNE-CiGBF3-F(EcoRⅠ) | ATGGGATCTACTAGTGAATTCATGGGGAACAATGAAGATGGA |
| L101YNE-CiGBF3-R(KpnⅠ) | TGGGTACATCCCGGGGGTACC GCCTGCAGCCACGGCA |
| JW771-CiGBF3-F(KpnⅠ) | ACGGGGGACGAGCTCGGTACCATGGGGAACAATGAAGATGGA |
| JW771-CiGBF3-R(SalⅠ) | CGCGTACGAGATCTGGTCGAC GCCTGCAGCCACGGCA |
| JW772-CiNFYA1-F(KpnⅠ) | TACGCGTCCCGGGGCGGTACC ATGTTACGAAGCATGC |
| JW772-CiNFYA1-R(SalⅠ) | ACGAAAGCTCTGCAGGTCGAC TCATTTGATGGTCAGG |
