# Supplementary material for: The Transcription Factors NFYA1 and GBF3 Jointly Regulate CHS2 to Promote Tangeretin Accumulation and Cold Tolerance in Citrus
Source: Plant Biotechnol J. 2025 Sep 16;24(2):582–601. doi: 10.1111/pbi.70371 (PMC12906813; doi:10.1111/pbi.70371)
Supplement: Supplementary file 2 — Figure S1: The relative tangeretin content of HB pummelo (HB) and Ichang papeda (Yi Chang Cheng, YCC) under cold treatment. Figure S2: Influence of different tangeretin concentrations on cold tolerance of HB pummelo. Figure S3: Exogenous tangeretin application enhanced cold tolerance of lemon. Figure S4: Molecular identification of the CiCHS2‐VIGS plants. Figure S5: Molecular identification of the CiCHS2‐overexpression plants. Figure S6: Overexpression of CiCHS2 enhanced cold tolerance of transgenic tobacco. Figure S7: Partial PCR identification results of Y1H screening. Figure S8: Expression patterns of CiNFYA1 and CiGBF3 under cold treatment. Figure S9: Phylogenetic analysis of CiNFYA1 and all NF‐Y transcription factors from Arabidopsis. Figure S10: Phylogenetic analysis of CiGBF3 and all bZIP transcription factors from Arabidopsis. Figure S11: Subcellular localization and transcriptional activation activity of CiGBF3 and CiNFYA1. Figure S12: Molecular identification of the CiGBF3‐overexpression plants. Figure S13: The expression levels of CiCHS2 in overexpressing‐CiGBF3 plants. Figure S14: Molecular identification of the TRV2‐CiGBF3 plants. Figure S15: The expression levels of CiCHS2 in TRV‐CiGBF3 plants. Figure S16: Molecular identification of the TRV2‐CiNFYA1 plants. Figure S17: The expression levels of CiCHS2 in TRV‐CiNFYA1 plants. Figure S18: The expression levels of CiGBF3 and CiNFYA1 in TRV:00 and VIGS plants. Figure S19: Expression analysis of COMTs in Ichang papeda under cold treatment. Figure S20: The expression of CiNFYA1, CiGBF3 and CiCHS2 in response to ABA. [file PBI-24-582-s002.docx]

Supplemental Figures

**The transcription factors NFYA1 and GBF3 jointly regulate *CHS2* to promote tangeretin accumulation and cold tolerance in *Citrus***


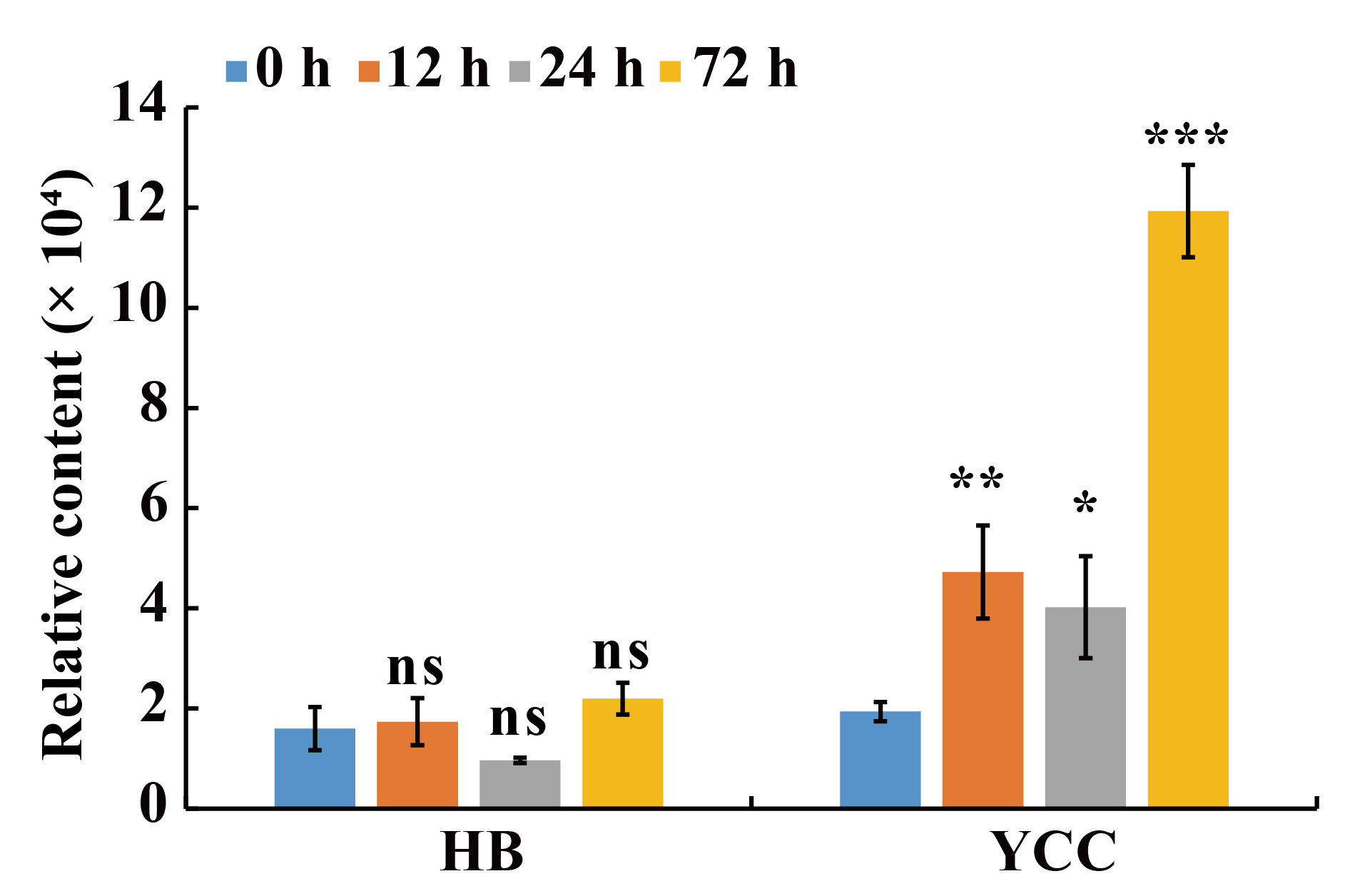


**Supplemental Figure S1. The relative tangeretin content of HB pummelo (HB) and Ichang papeda (Yi Chang Cheng, YCC) under cold treatment.**

Error bars indicate ± SD (n=3). Asterisks indicate that the values are significantly different from that of 0 h (based on the Student’s t-test: **P*< 0.05, ***P*< 0.01, ****P*< 0.001; ns, no significance).


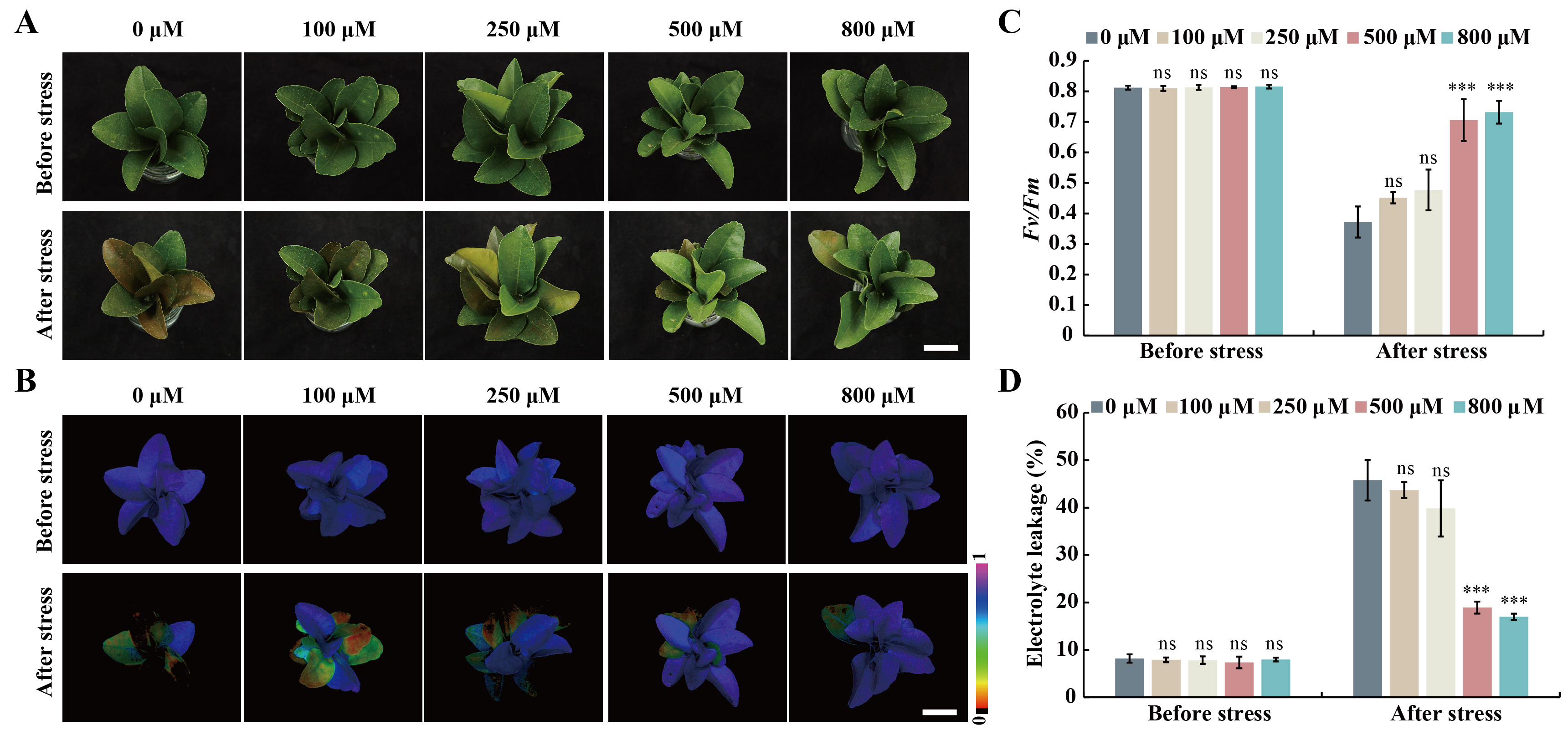


**Supplemental Figure S2. Influence of different tangeretin concentrations on cold tolerance of HB pummelo**

A, Phenotypes of HB pummelo shoots pretreated with tangeretin at different concentrations. Tangeretin was dissolved in methanol (10%, v/v) before and after cold stress. B-D, Chlorophyll fluorescence imaging (B), *Fv/Fm* value (C), and electrolyte leakage (D) of all tested plants before and after cold treatment. Asterisks indicate that the values are significantly different from that of 0 μM (based on the Student’s *t*-test: ****P*< 0.001; ns, no significance).


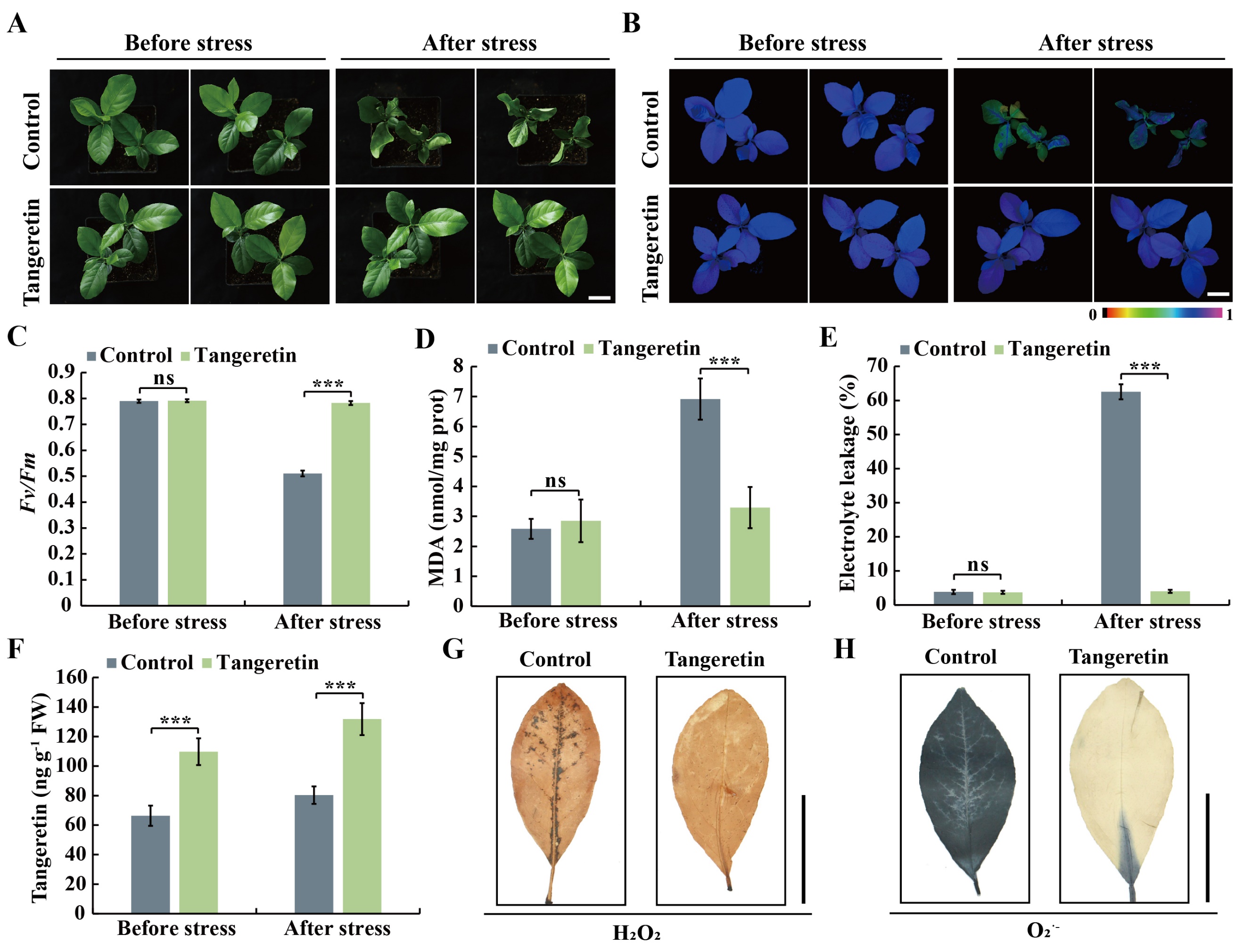


**Supplemental Figure S3. Exogenous tangeretin application enhanced cold tolerance of lemon.**

A-C, Phenotypes (A), chlorophyll fluorescence imaging (B) and *Fv/Fm* value (C) of lemon seedlings pretreated with or without 500 μM tangeretin before and after cold stress. Tangeretin was dissolved in methanol (10%, v/v). D-F, MDA content (D), electrolyte leakage (E), and tangeretin content (F) of all tested plants before and after cold treatment. G-H, Histochemical staining with 3,3-diaminobenzidine (DAB, G) and nitro blue tetrazolium (NBT, H) for detection of *in situ* accumulation of H_2_O_2_ and O_2_^•-^, respectively, in the leaves. Images in (G) and (H) were digitally extracted for comparison. Scale Bars = 3 cm. FW, fresh weight. Error bars represent ± SD (n = 3). Asterisks indicate that the values are significantly different between the involved pairs (based on the Student’s *t*-test: ****P*< 0.001; ns, no significance).


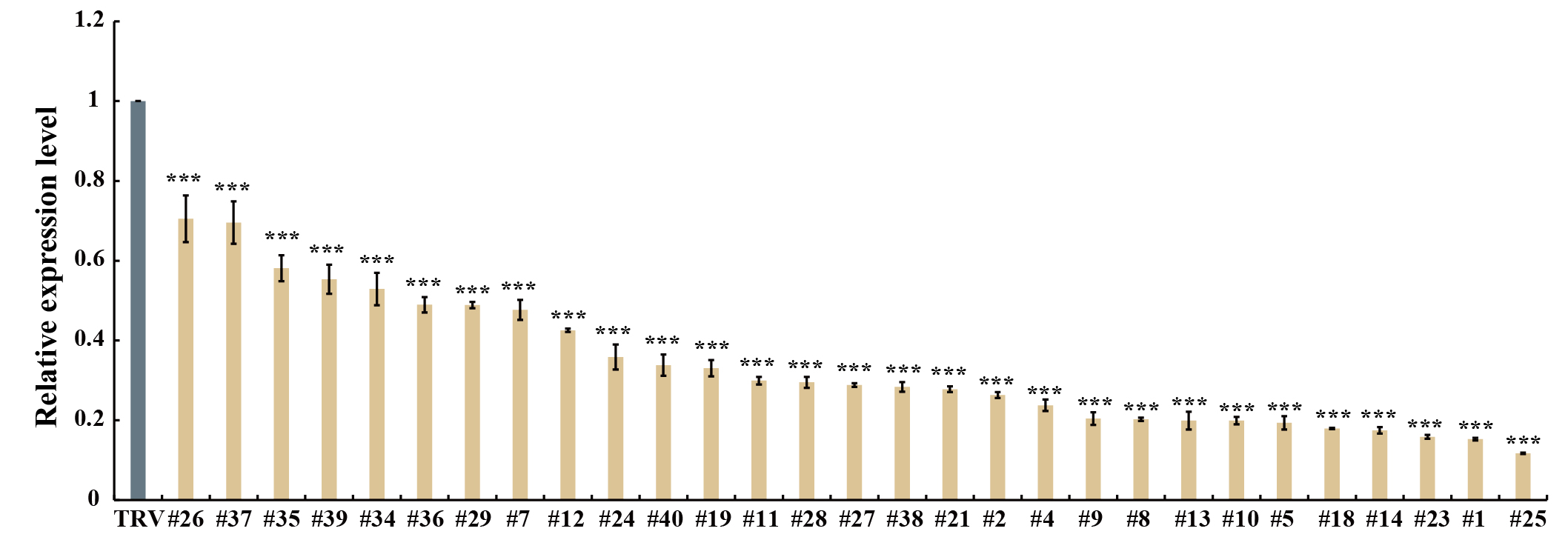


**Supplemental Figure S4. Molecular identification of the *CiCHS2*-VIGS plants.**

RT-qPCR was used to analyze the *CiCHS2* expression of TRV2-*CiCHS2* plants. *Actin* was used as an internal control. Error bars indicate ± SD (n = 3). Asterisks indicate that the values are significantly different from that of TRV (based on the Student’s t-test: ****P*< 0.001).


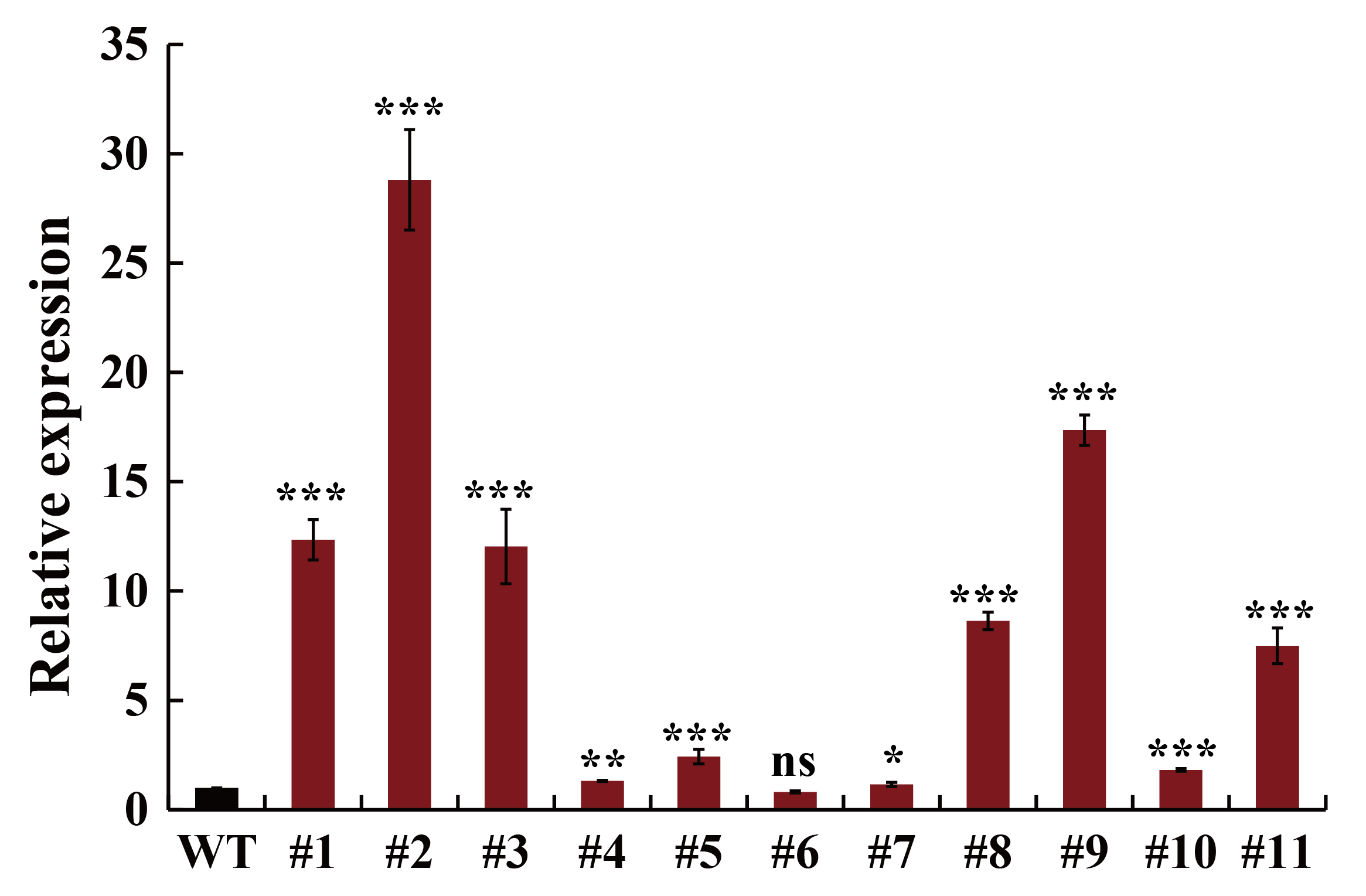


**Supplemental Figure S5. Molecular identification of the *CiCHS2*-overexpression plants.**

RT-qPCR was used to analyze the *CiCHS2* expression of *CiCHS2-*overexpression plants. *Actin* was used as an internal control. WT, wild type. Error bars indicate ± SD (n = 3). Asterisks indicate that the values are significantly different between the involved pairs (based on the Student’s *t*-test: **P*< 0.05, ***P*< 0.01, ****P*< 0.001; ns, no significance, *P*>0.05).


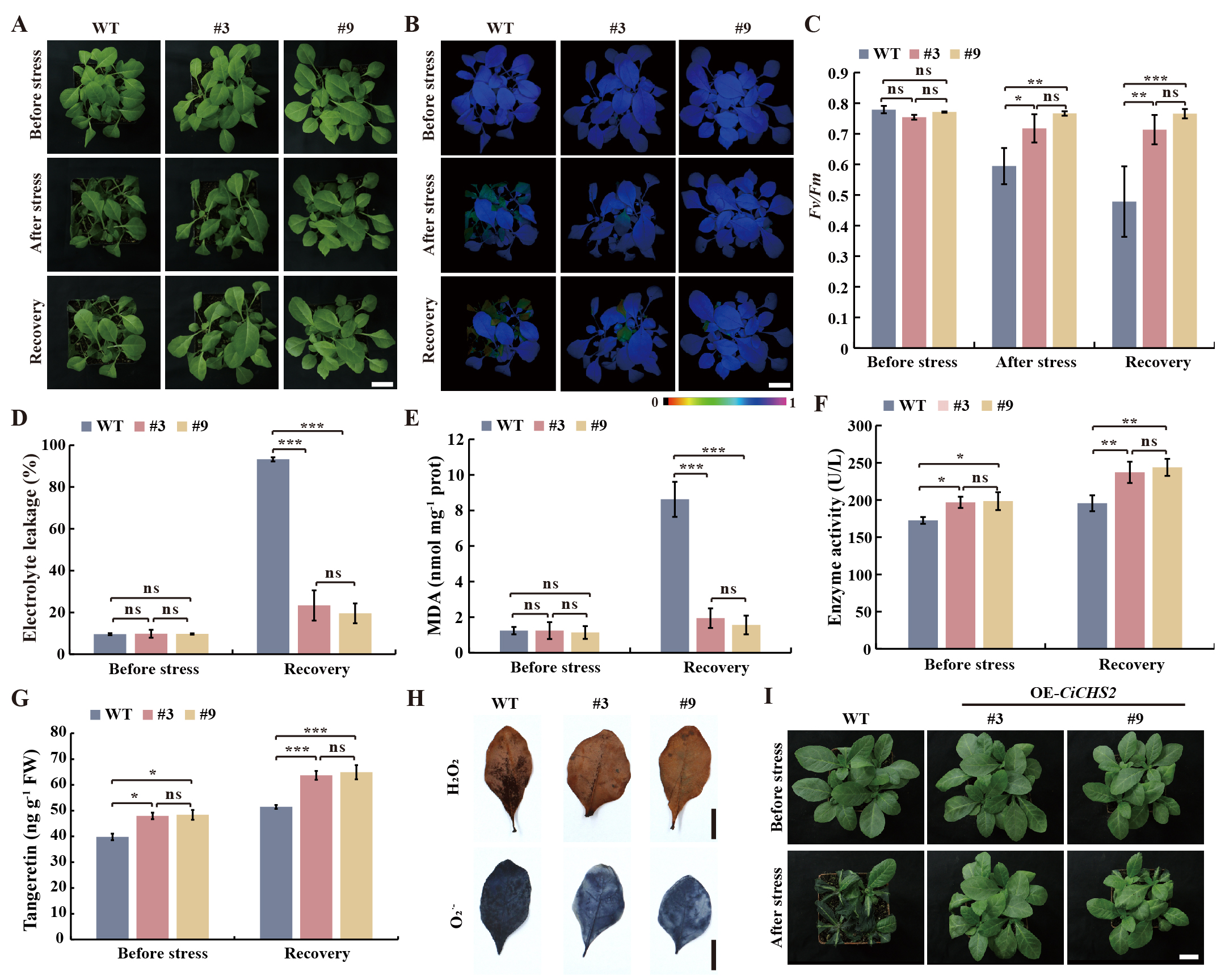


**Supplemental Figure S6. Overexpression of *CiCHS2* enhanced cold tolerance of transgenic tobacco.**

A-C, Phenotypes (A), chlorophyll fluorescence (B) and *Fv/Fm* ratios (C) of four-week-old transgenic lines (#3 and #9) and wild-type (WT) before, after cold treatment and subsequent growth recovery for 1 d at 25 °C. The false color scale is shown below the imaging. D-G, Electrolyte leakage (D), MDA contents (E), CHS activity (F), and tangeretin contents (G) of the tested lines before and after the growth recovery. H, Histochemical staining with 3, 3-diaminobenzidine (DAB, upper panel) and nitro blue tetrazolium (NBT, bottom panel) for detection of *in situ* accumulation of H_2_O_2_ and O_2_^•−^, respectively, in the transgenic plants and WT after cold treatment. I, Phenotypes of 7-week-old transgenic and WT plants after the cold treatment. Scale bars = 3 cm. FW, fresh weight. Error bars indicate ± SD (n = 3). Asterisks indicate that the values are significantly different between the involved pairs (based on the Student’s *t*-test; **P* < 0.05, ***P*< 0.01, ****P*< 0.001; ns, no significance).





**Supplemental Figure S7. Partial PCR identification results of Y1H screening.**

The Y1H positive yeast colonies were identified by PCR. M: DNA marker.


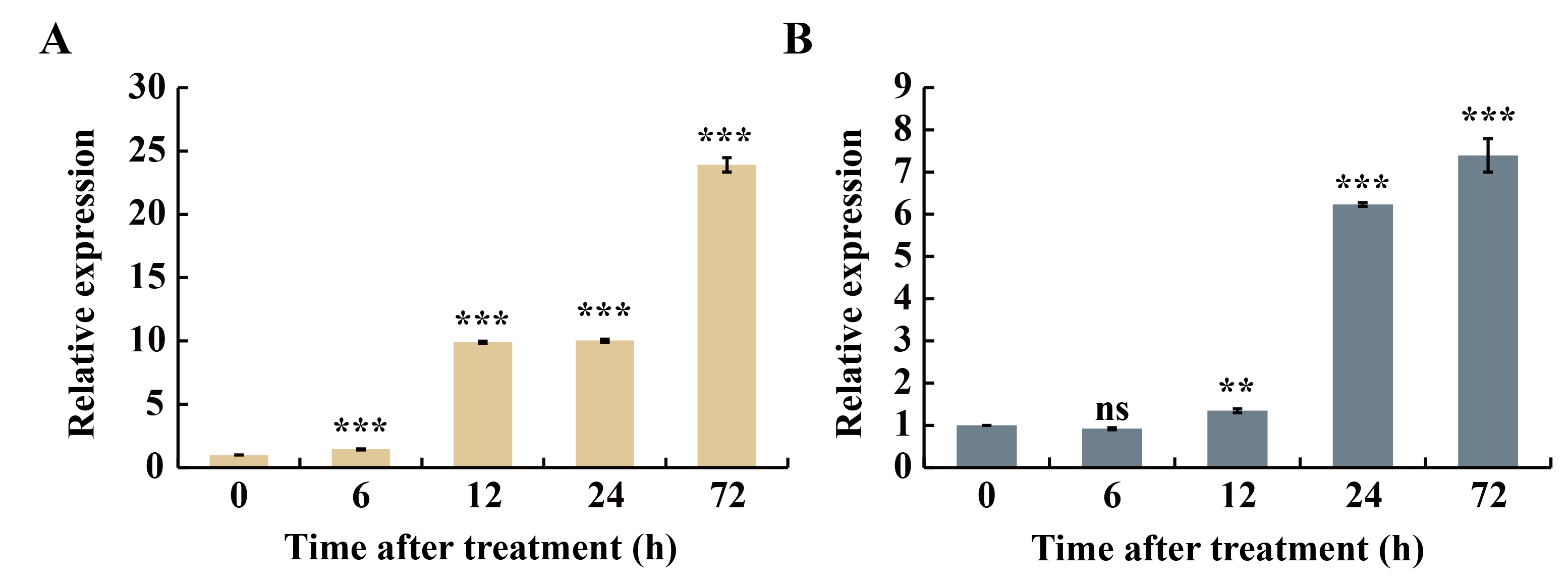


**Supplemental Figure S8. Expression patterns of *CiNFYA1* and *CiGBF3* under cold treatment.**

A-B, Relative expression levels of *CiNFYA1* (A) and *CiGBF3* (B) in Ichang papeda under cold treatment (4 °C) by using RT-qPCR. *Actin* was used as an internal control. Error bars indicate ± SD (n=3). Asterisks indicate that the values are significantly different from that of 0 h (based on the Student’s *t*-test; ***P*< 0.01, ****P*< 0.001; ns, no significance, *P*>0.05).

**
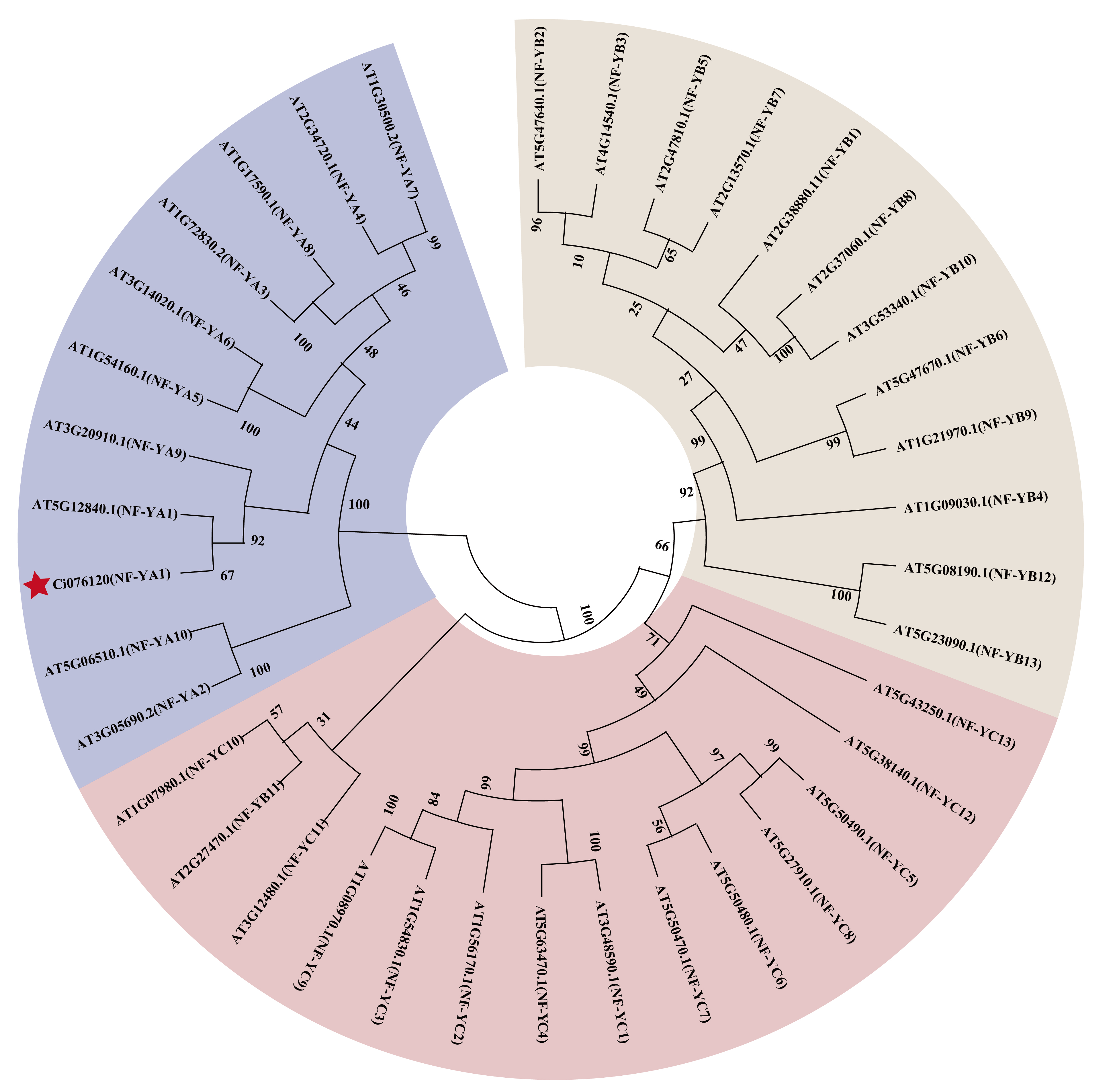
**

**Supplemental Figure S9. Phylogenetic analysis of CiNFYA1 and all NF-Y transcription factors from *Arabidopsis*.**

The phylogenetic tree was constructed with full-length amino acid sequences of CiNFYA1 from Ichang papeda (*Citrus ichangensis*) and NF-Y proteins from *Arabidopsis thaliana* by the Maximum Likelihood method in the default parameter using MEGAX software.


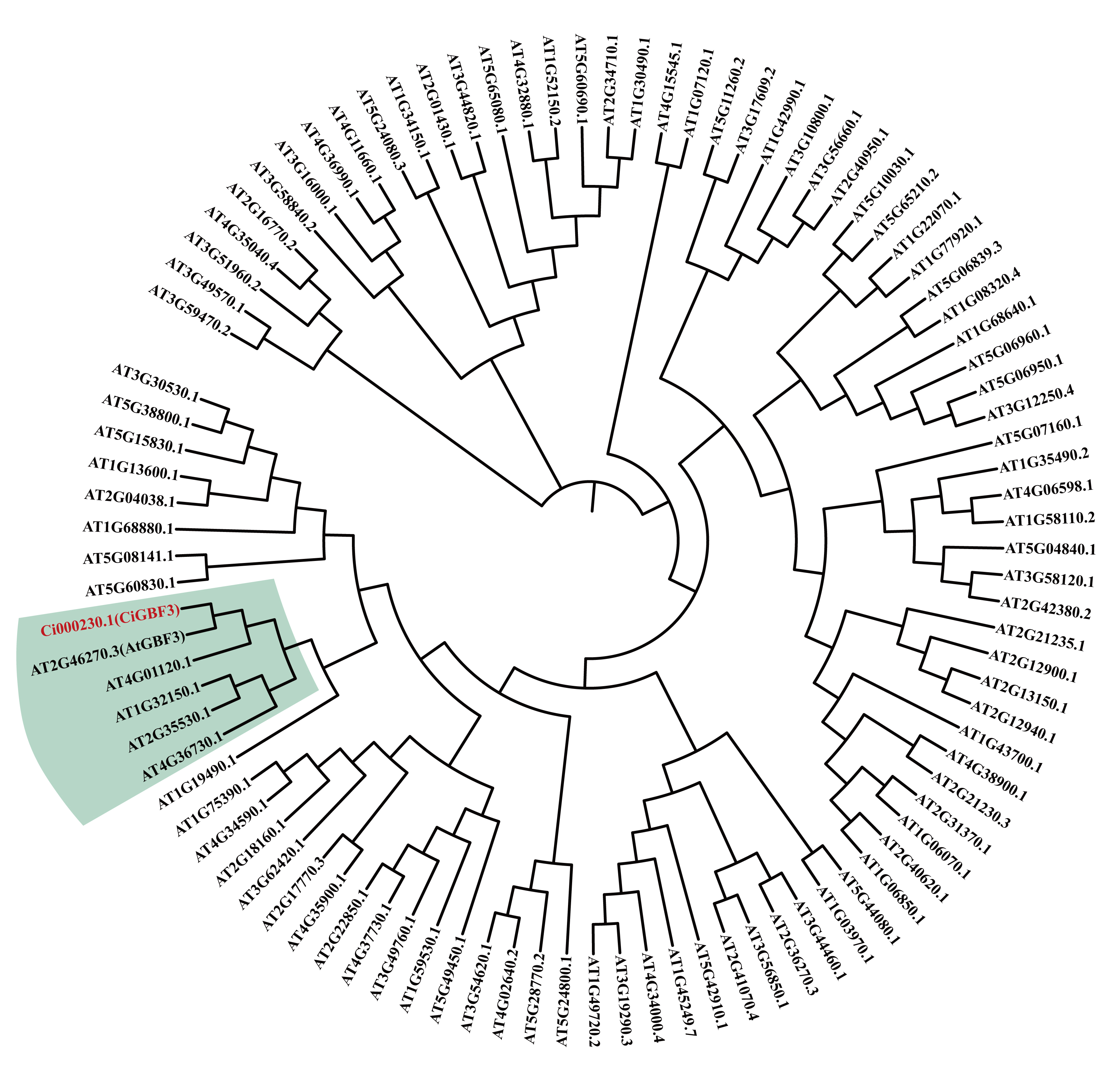


**Supplemental Figure S10. Phylogenetic analysis of CiGBF3 and all bZIP transcription factors from *Arabidopsis*.**

The phylogenetic tree was constructed with full-length amino acid sequences of CiGBF3 from Ichang papeda (*Citrus ichangensis*) and all bZIP family proteins from *Arabidopsis thaliana* by the Maximum Likelihood method in the default parameter using MEGAX software.


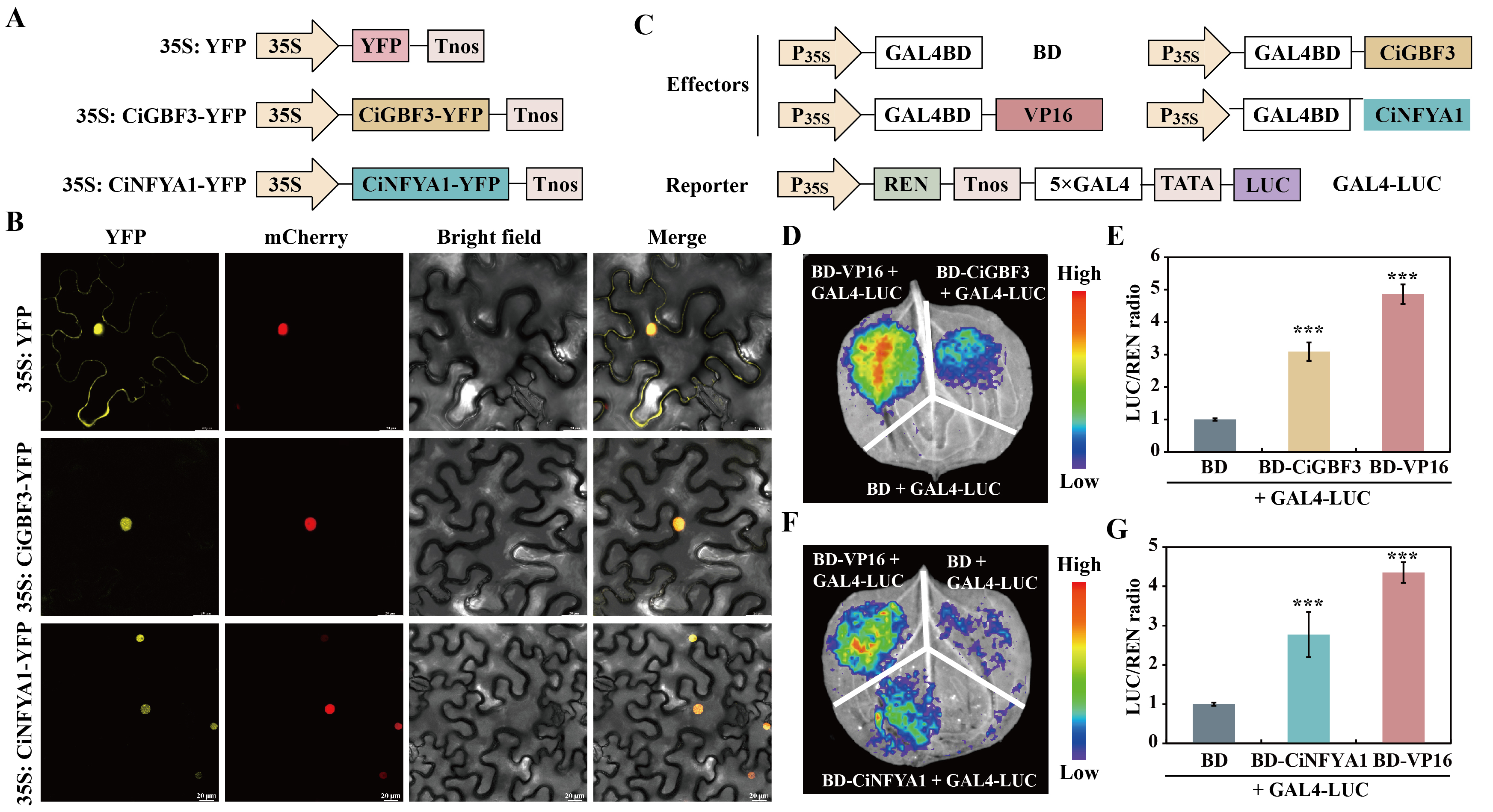


**Supplemental Figure S11. Subcellular localization and transcriptional activation activity of CiGBF3 and CiNFYA1.**

A, Schematic diagram of the vectors used for subcellular localization; B, Confocal laser microscopy images showing the mcherry and CiGBF3-YFP and CiNFYA1-YFP fluorescence signals in tobacco epidermal cells from transiently transformed *Nicotiana benthamiana* leaves (scale bars = 20 μm). C, Schematic diagrams of reporter and effector constructs used for transcriptional activation activity analysis of CiGBF3 and CiNFYA1 *in vivo*. BD-VP16 and BD were used as positive and negative controls, respectively. D-G, Luciferase (LUC) fluorescence imaging (D, F) and LUC/REN ratio (E, G) in tobacco leaves co-infiltrated with the indicated vectors. Images in (D and F) were digitally extracted for comparison. Error bars indicate ±SD (n = 3). Asterisks indicate that the values are significantly different from that of the negative control (based on the Student’s *t*-test: ***P< 0.001).


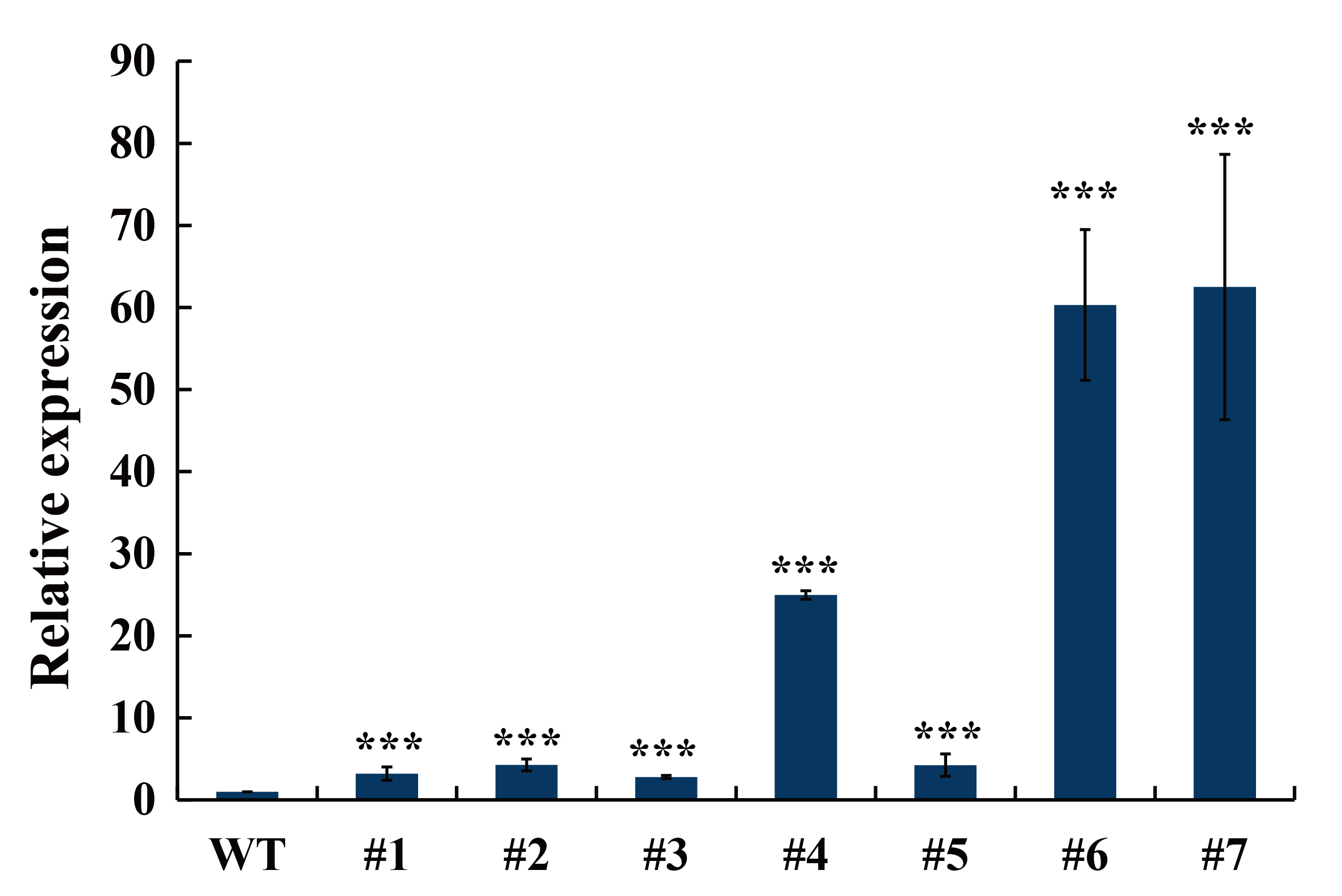


**Supplemental Figure S12. Molecular identification of the *CiGBF3*-overexpression plants.**

RT-qPCR was used to analyze the *CiGBF3* expression of *CiGBF3-*overexpression plants. *Actin* was used as an internal control. WT, wild type. Error bars indicate ± SD (n = 3). Asterisks indicate that the values are significantly different from that of the WT (based on the Student’s *t*-test: ***P< 0.001).


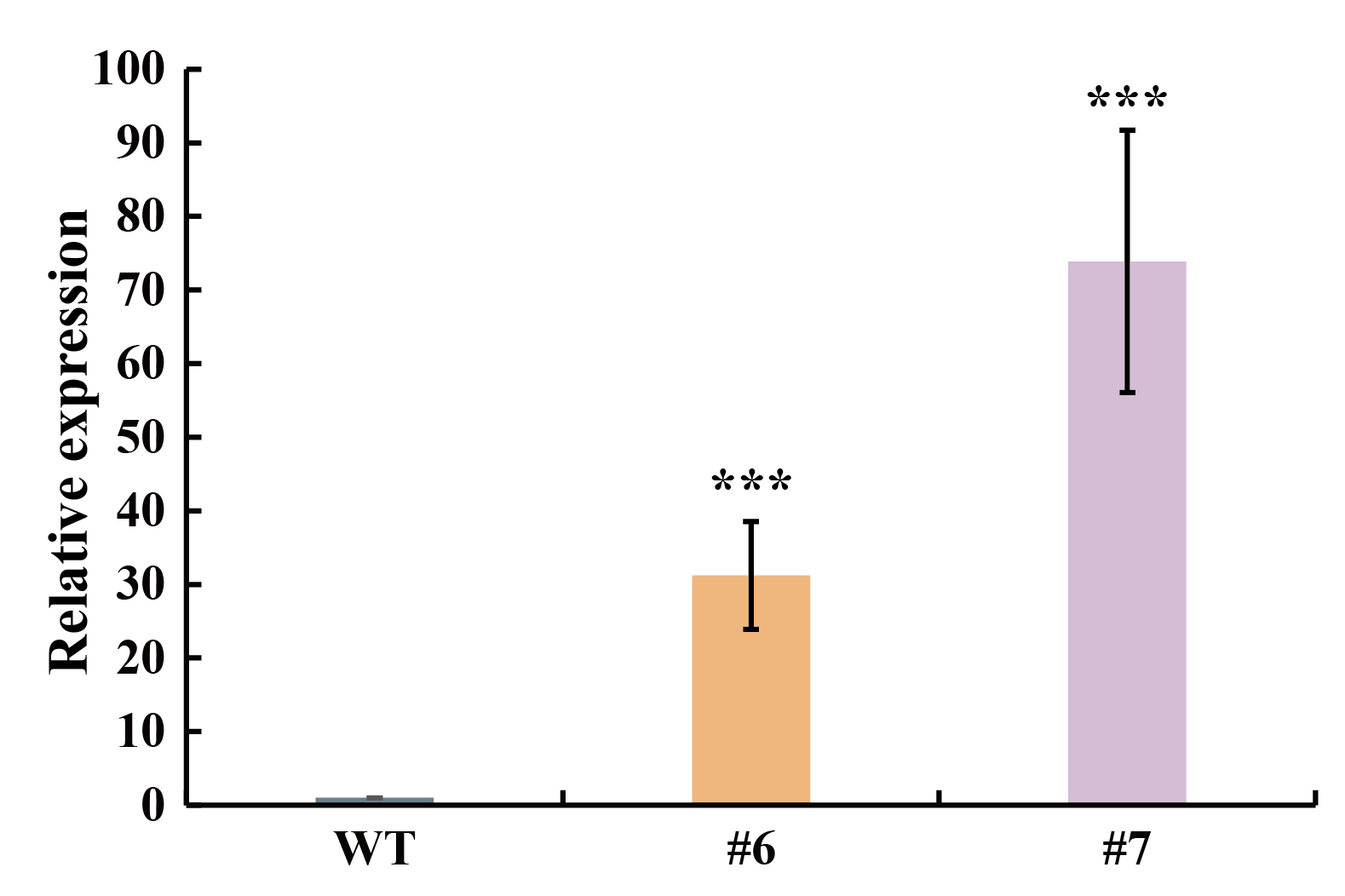


**Supplemental Figure S13.** **The expression levels of *CiCHS2* in overexpressing**-***CiGBF3* plants.**

RT-qPCR was used to analyze the *CiCHS2* expression of overexpression-*CiGBF3* plants. *Actin* was used as an internal control. WT, wild type. Error bars indicate ± SD (n = 3). Asterisks indicate that the values are significantly different from that of WT (based on the Student’s t-test: ****P*< 0.001).


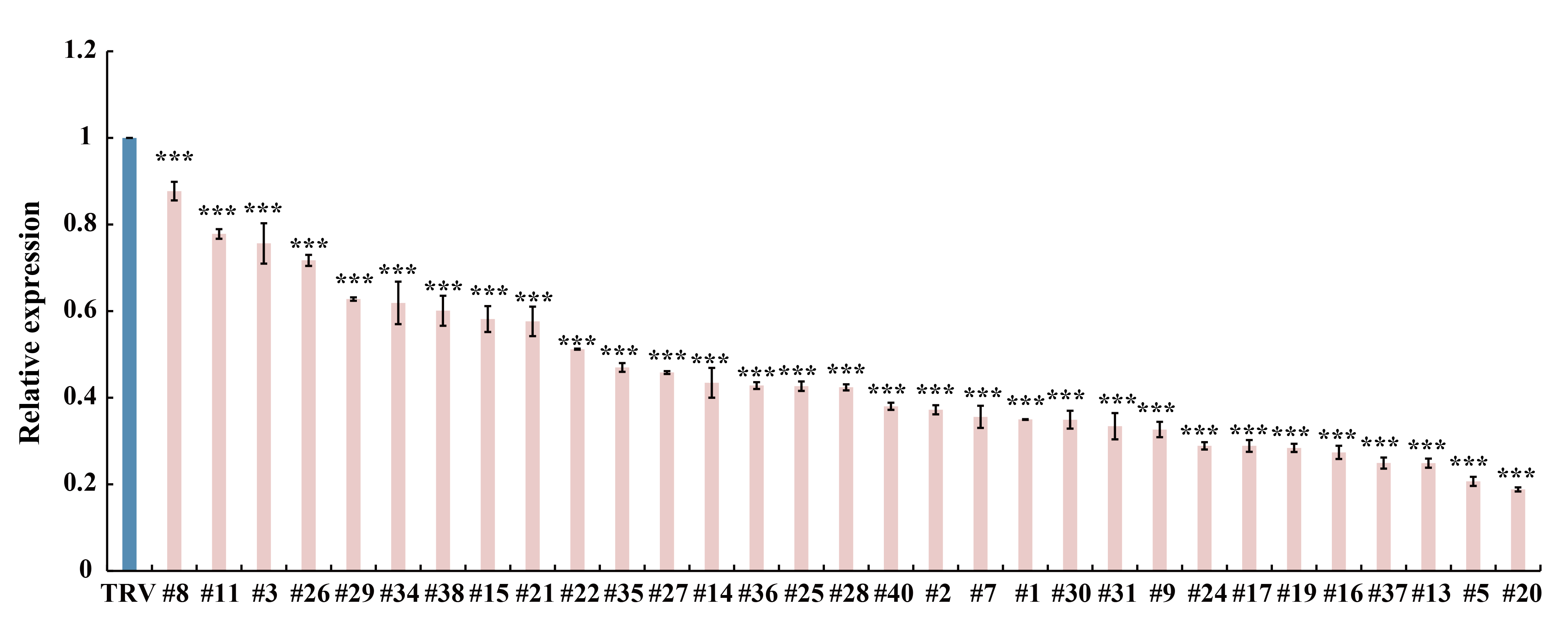


**Supplemental Figure S14.** **Molecular identification of the TRV2**-***CiGBF3* plants.**

RT-qPCR was used to analyze the *CiGBF3* expression of TRV2-*CiGBF3* plants. *Actin* was used as an internal control. Error bars indicate ± SD (n = 3). Asterisks indicate that the values are significantly different from that of TRV (based on the Student’s t-test: ****P*< 0.001).


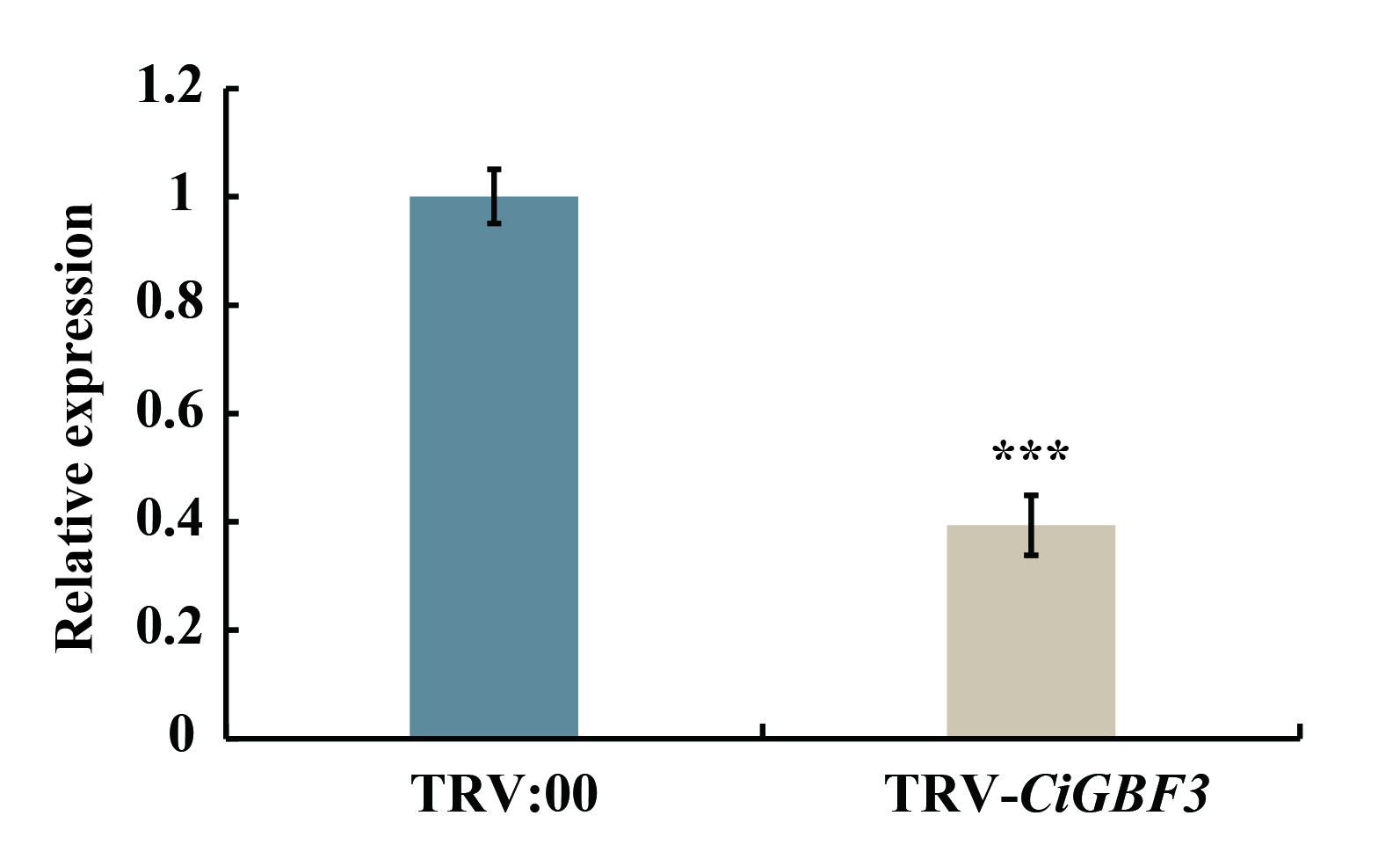


**Supplemental Figure S15.** **The expression levels of *CiCHS2* in TRV-*CiGBF3* plants.**

RT-qPCR was used to analyze the *CiCHS2* expression of TRV2-*CiGBF3* plants. *Actin* was used as an internal control. Error bars indicate ± SD (n = 3). Asterisks indicate that the values are significantly different from that of TRV:00 control (based on the Student’s t-test: ****P*< 0.001).

**
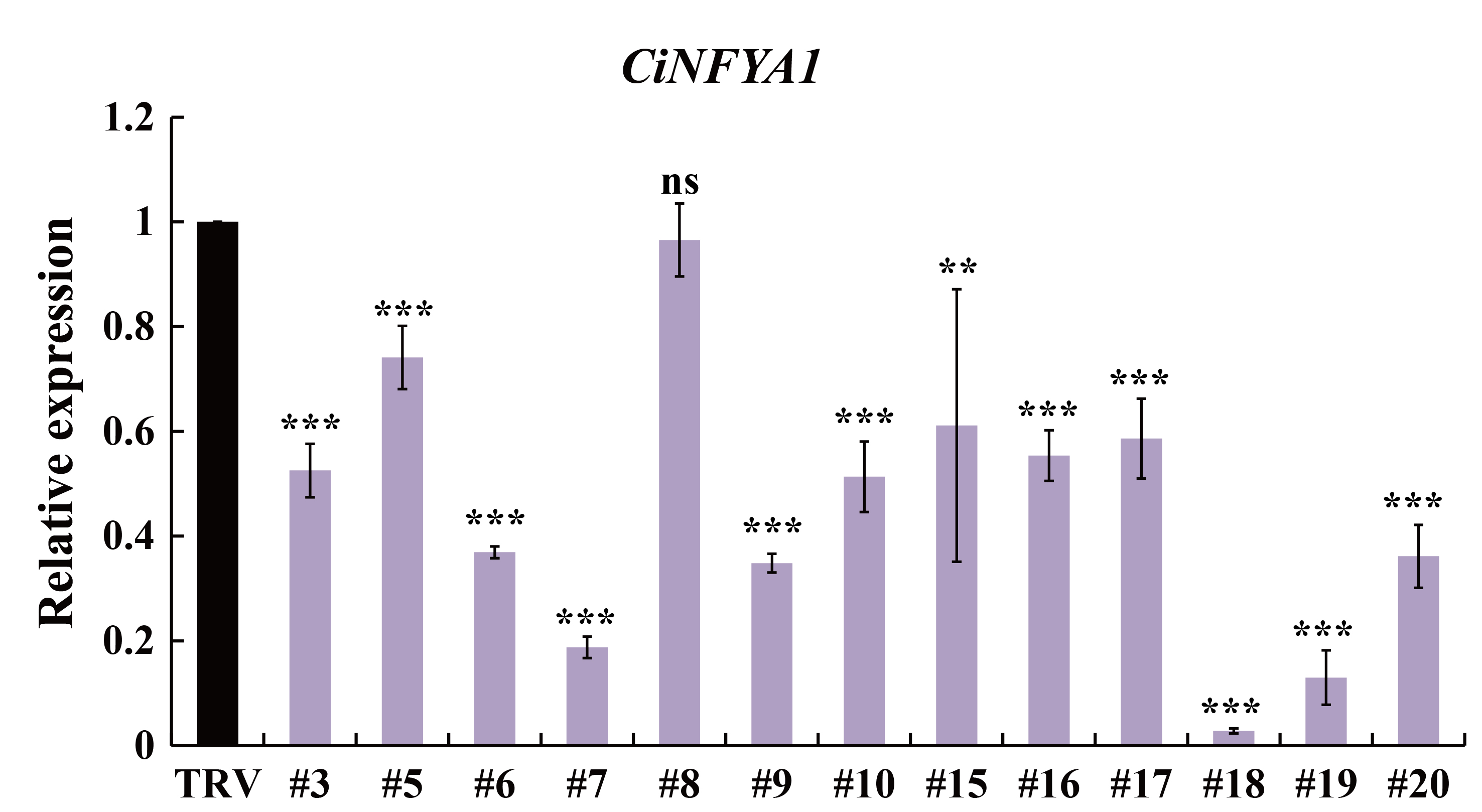
**

**Supplemental Figure S16.** **Molecular identification of the TRV2-*CiNFYA1* plants.**

RT-qPCR was used to analyze the *CiNFYA1* expression of TRV2-*CiNFYA1* plants. *Actin* was used as an internal control. Error bars indicate ± SD (n = 3). Asterisks indicate that the values are significantly different from that of TRV (based on the Student’s t-test: ***P*< 0.01, ****P*< 0.001; ns, no significance).


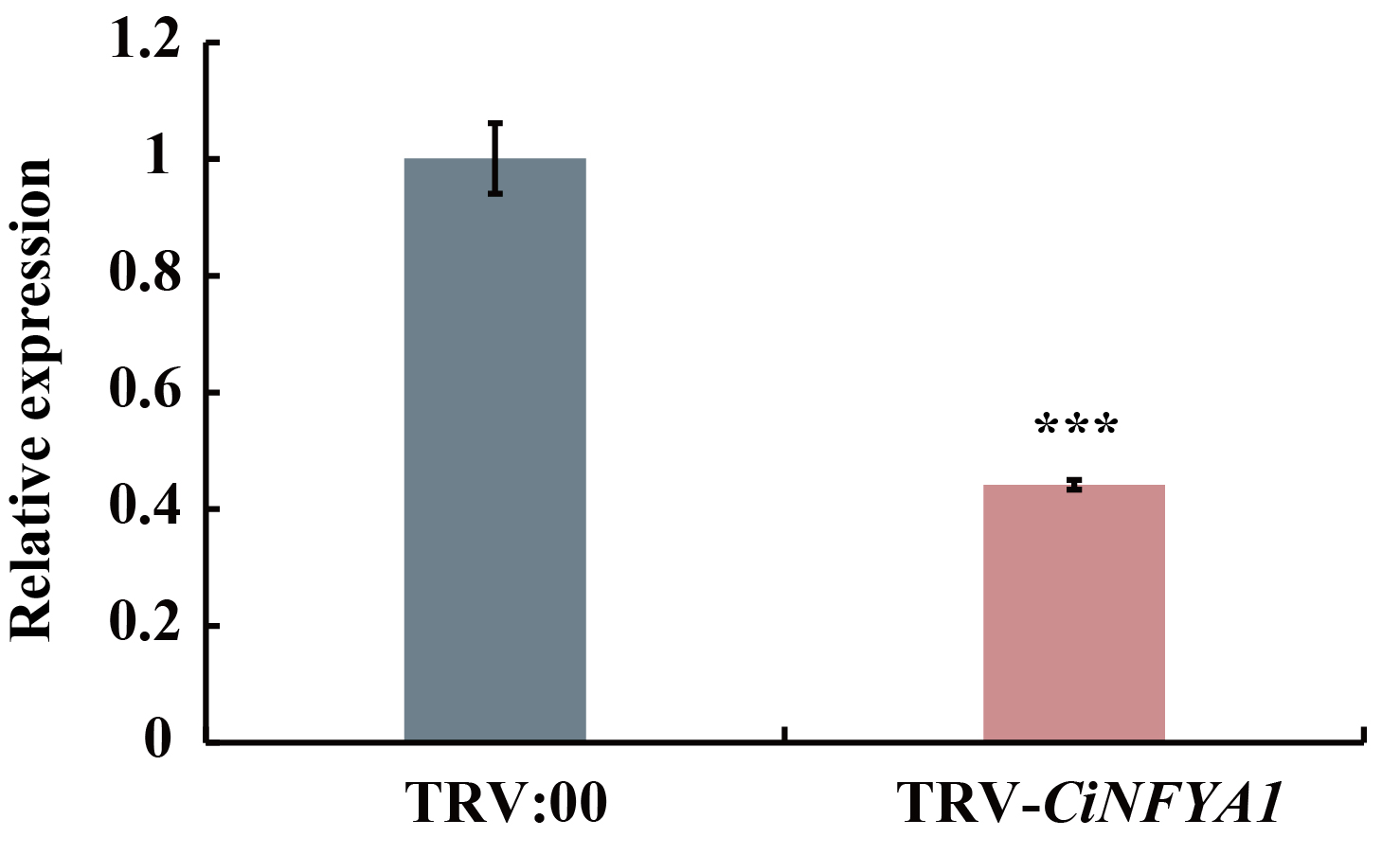


**Supplemental Figure S17.** **The expression levels of *CiCHS2* in TRV-*CiNFYA1* plants.**

The expression levels of *CiCHS2* in TRV-*CiNFYA1* plants were detected by RT-qPCR. *Actin* was used as an internal control. Error bars indicate ± SD (n = 3). Asterisks indicate that the values are significantly different from that of TRV:00 control (based on the Student’s t-test: ****P*< 0.001).


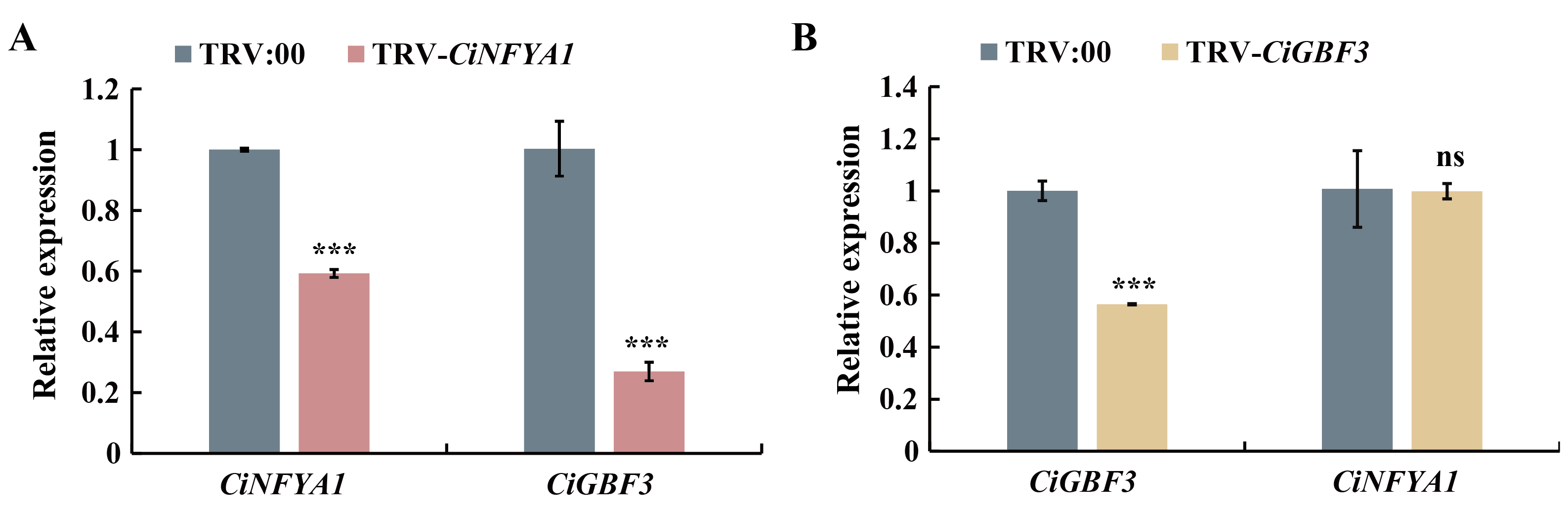


**Supplemental Figure S18. The expression levels of *CiGBF3* and *CiNFYA1* in TRV:00 and VIGS plants.**

The expression levels of *CiNFYA1* and *CiGBF3* in TRV-*CiNFYA1* plants (A) and in TRV-*CiGBF3* plants (B) were detected by RT-qPCR. *Actin* was used as an internal control. Error bars indicate ± SD (n = 3). Asterisks indicate that the values are significantly different from that of TRV:00 control (based on the Student’s t-test: ****P*< 0.001; ns, no significance).

**
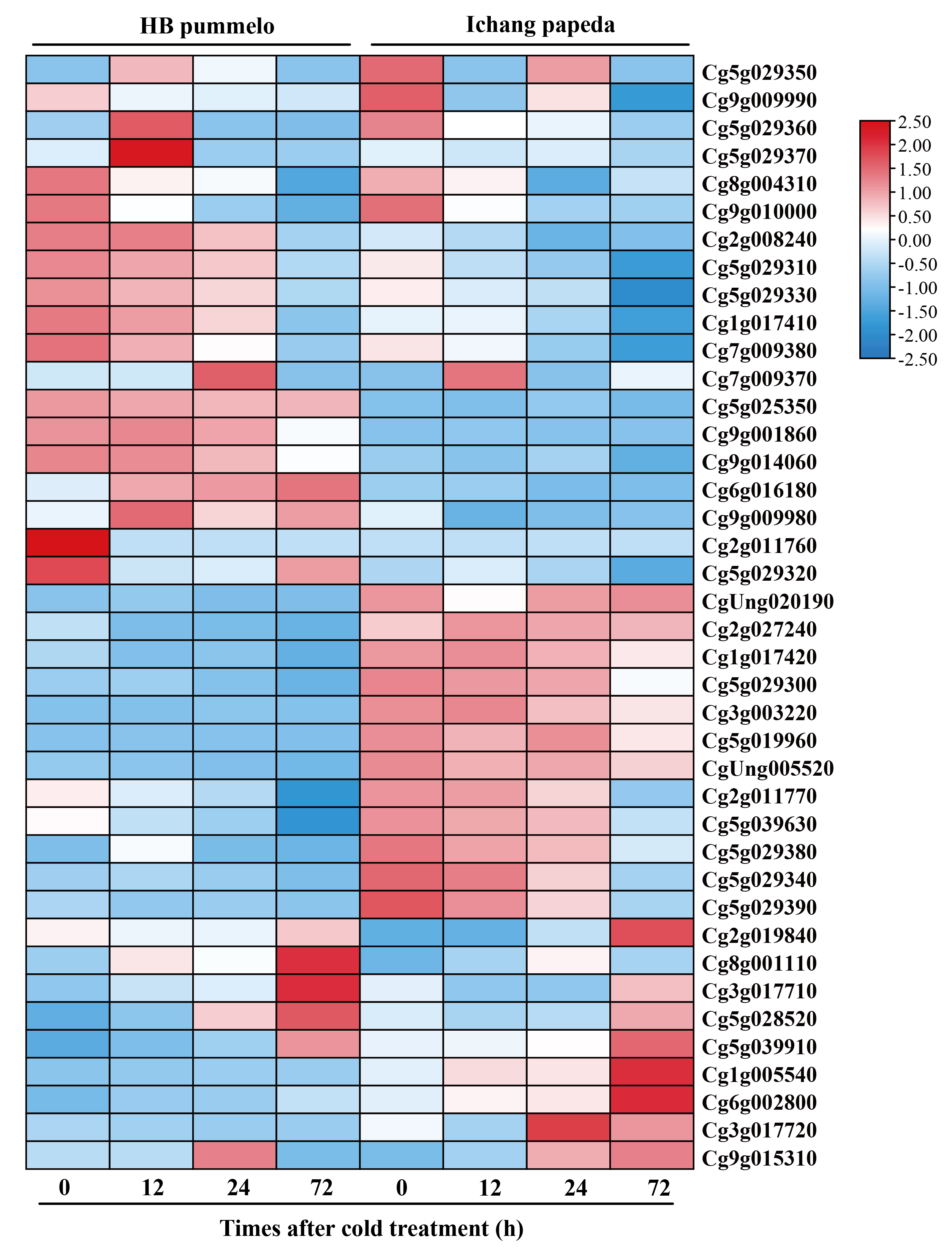
**

**Supplemental Figure S19.** **Expression analysis of *COMTs* in Ichang papeda under cold treatment.**

Comparative analysis of the transcript levels of *COMTs* in HB pummelo and Ichang papeda under cold treatment. The colored bar indicates log_2_ (fold-change) values.

**
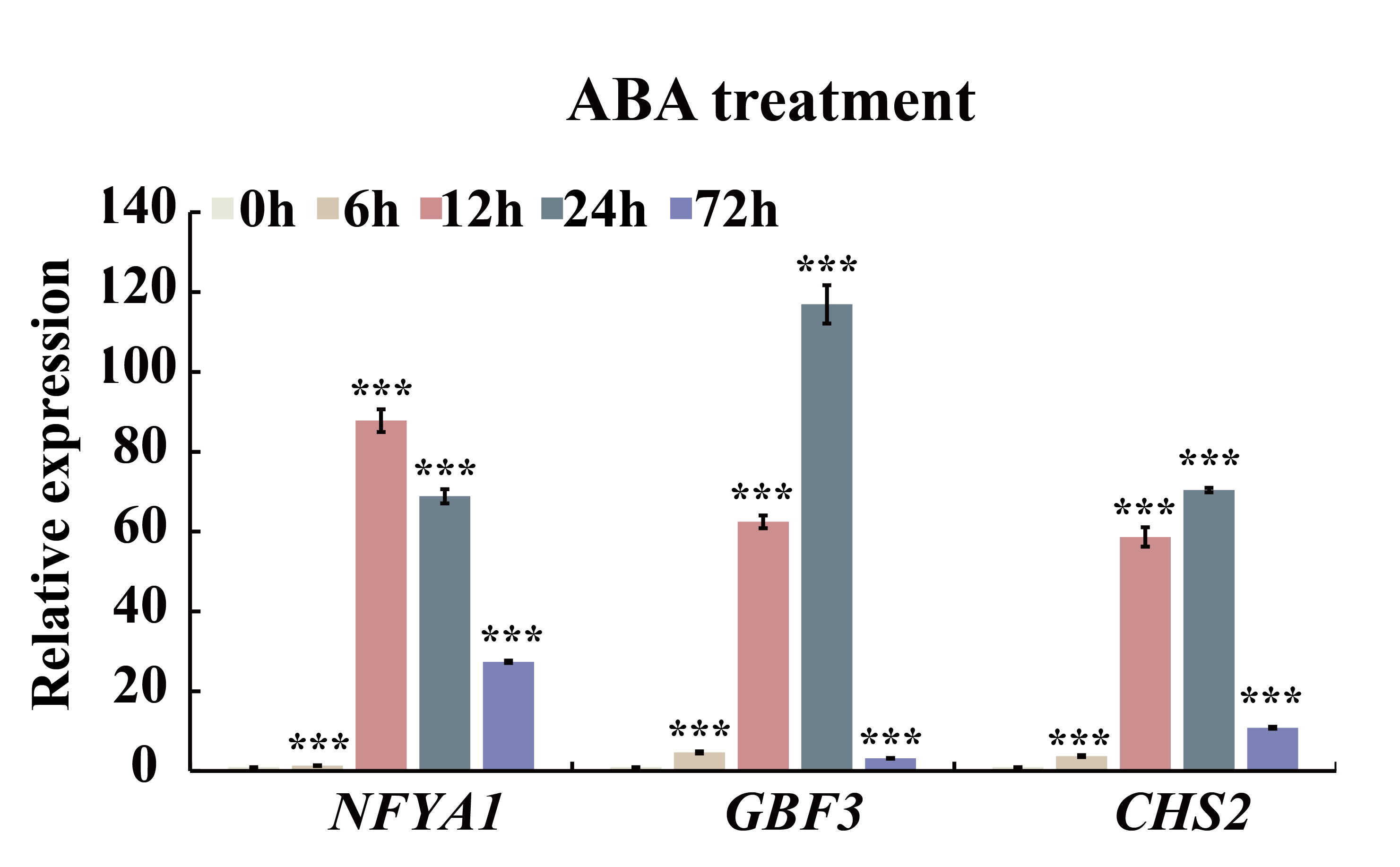
**

**Supplemental Figure S20. The expression of *CiNFYA1*, *CiGBF3 and CiCHS2* in response to ABA.**

The expression of *CiNFYA1*, *CiGBF3 and CiCHS2* under ABA (10 mM) treatment was detected by RT-qPCR. *Actin* was used as an internal control. Error bars indicate ± SD (n = 3). Asterisks indicate that the values are significantly different from that of 0 h (based on the Student’s *t*-test; ****P*< 0.001).
